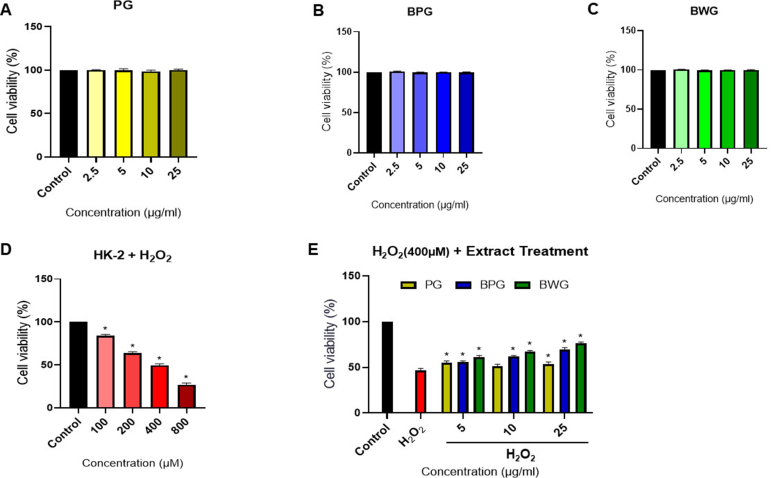# Renoprotective mechanisms of bioconverted wild-simulated ginseng: mitigating oxidative stress, inflammation, and apoptosis to protect against ischemic renal injury via Nrf2/HO-1/NF-κB/caspase-3 signaling

**DOI:** 10.1016/j.jgr.2025.10.007

**Published:** 2025-10-26

**Authors:** Ye Ji Kim, Md Shiblee Sadik Sabuj, Myung-Kon Kim, Joonseok Lee, Ryunhee Kim, Seung Hyun Lee, Jin Min Oh, Hyeon Gyeong Ro, In-Shik Kim, Dongchoon Ahn, Md Rashedunnabi Akanda, Hyun-Jin Tae, Byung-Yong Park

**Affiliations:** a College of Veterinary Medicine and Institute of Animal Transplantation, Jeonbuk National University, Iksan, 54596, Republic of Korea; b Department of Food Science and Technology, Jeonbuk National University, Jeonju, 54896, Republic of Korea; c Department of Pharmacology and Toxicology, Sylhet Agricultural University, Sylhet, 3100, Bangladesh

**Keywords:** Bioconverted wild-simulated ginseng, Ischemic renal injury, Nrf2/HO-1/NF-κB/Caspase-3 signaling, Oxidative stress, Renoprotection

## Abstract

**Background:**

Ischemia-reperfusion (I/R) induced acute kidney injury (AKI) is a severe condition linked to higher morbidity and mortality. *Panax ginseng* (PG) possesses renoprotective properties; however, its inadequate bioavailability restricts its efficacy. Wild-simulated PG (WSG), processed to boost its bioactive components, exhibits improved renoprotective effects. This research examines PG, bioconverted PG (BPG), and bioconverted WSG (BWG) for their protective roles against I/R induced AKI in mice.

**Methods:**

C57BL/6 mice underwent bilateral renal pedicle clamping via a dorsal approach for 30 min to induce ischemia, followed by clamp release and 24 h of reperfusion. Blood and kidney samples were collected at the end of the reperfusion period. Sham-operated mice underwent identical procedures without vascular clamping. Additionally, hydrogen peroxide (H_2_O_2_) induced oxidative stress in HK-2 cells.

**Results:**

The effects of each extract were evaluated. Expression of antioxidant, inflammatory, and apoptotic factors was confirmed using western blotting, immunohistochemistry, and TUNEL assays. Both extracts improved the survival of H_2_O_2_-treated HK-2 cells and enhanced kidney function, as indicated by reduced BUN and Cr levels. Histopathological analysis showed decreased injury, preserved tubular structure, and reduced inflammation in extract-treated groups. These protective effects were linked to activation of the Nrf2/HO-1 pathway, leading to increased expression of antioxidant enzymes (CAT, GPX-1, SOD-1) and reduced malondialdehyde (MDA) levels. Moreover, the extracts downregulated pro-inflammatory cytokines (TNF-α, IL-1β), suppressed NF-κB phosphorylation, decreased Bax/Bcl-2 ratio and Caspase-3 activation, enhancing cell survival.

**Conclusion:**

BPG and BWG remarkably ameliorated I/R-induced AKI through modulation of Nrf2/HO-1/NF-κB/Caspase-3 signaling pathway, with BWG exhibiting more substantial renoprotective effects.

## Graphical abstract

**Figure:**
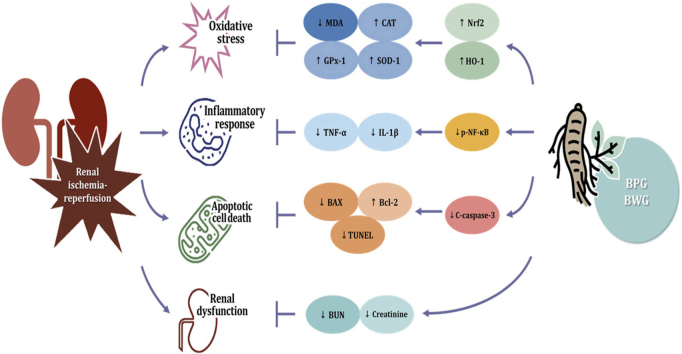

## 1 Introduction

Renal I/R injury is a major clinical challenge associated with AKI, often leading to chronic kidney disease and increased mortality [1,2]. The pathophysiology occurs due to a temporary disruption of blood flow to the kidneys, followed by reperfusion, which exacerbates tissue damage through oxidative stress, inflammatory responses, and apoptosis [3]. The imbalance between reactive oxygen species (ROS) generation and the antioxidant defense system leads to oxidative stress, while the activation of pro-inflammatory cytokines amplifies tissue injury and kidney dysfunction [4,5]. A significant contributor to I/R-induced damage is the excessive generation of ROS, which disrupts the cellular homeostasis, triggers lipid peroxidation, and results in mitochondrial dysfunction [[6], [7], [8]]. Antioxidant enzymes are crucial in mitigating oxidative stress and safeguarding renal cells against damage induced by I/R [9]. The vital antioxidant defense mechanisms, such as superoxide dismutase (SOD), catalase (CAT), glutathione peroxidase (GPX), and heme oxygenase-1 (HO-1), assist in neutralizing ROS, preserving cellular redox balance, and controlling inflammation [10].

A key mechanism underlying renal I/R injury is the activation of inflammatory pathways, particularly the upregulation of pro-inflammatory cytokines such as tumor necrosis factor-alpha (TNF-α), interleukin-6 (IL-6), and interleukin-1 beta (IL-1β) [11,12]. These cytokines contribute to renal injury by promoting leukocyte infiltration, endothelial dysfunction, and fibrosis [13,14]. Moreover, oxidative stress, like ROS, worsens inflammation, leading to a harmful cycle of kidney injury that increases the expression of several inflammatory markers [15]. In I/R injury, oxidative stress plays a crucial role in causing cellular damage. The Nrf2/HO-1 pathway serves as an essential antioxidant defense system, with Nrf2 activation triggering the expression of HO-1, which reduces ROS buildup, lessens lipid peroxidation, and preserves mitochondrial integrity [16]. In contrast, the equilibrium between pro-apoptotic Bax and anti-apoptotic Bcl-2 proteins determines cell survival during oxidative stress [17]. Ischemia usually elevates Bax expression and reduces Bcl-2, facilitating mitochondrial outer membrane permeabilization and apoptosis. The activation of Nrf2/HO-1 can indirectly decrease Bax levels while increasing Bcl-2 expression, leading to reduced apoptosis and enhanced renal cell survival following ischemic or oxidative damage [18]. A transcription factor, the Nrf2/HO-1 pathway, is crucial for cellular protection against oxidative stress, whereas NF-κB is a significant mediator of inflammation during renal I/R injury [19,20]. In the renal I/R injury, the equilibrium between Bax and Bcl-2 is disrupted. An alteration in the Bax/Bcl-2 ratio may increase cell death and advance renal I/R injury [21]. Consequently, altering the Bax/Bcl-2 ratio may trigger the transcription factor caspase-3 and influence apoptosis in experimental I/R induced AKI [22]. Therefore, apoptosis-related proteins, like Caspase-3, play a vital role in regulating renal cell survival in ischemic injury [23]. Altering these signaling pathways could offer a hopeful approach to kidney protection.

Ginseng, a well-known medicinal plant, has been historically used for its various therapeutic properties, including antioxidant, anti-inflammatory, and anti-apoptotic effects [[24], [25], [26], [27], [28]]. PG denotes the traditional variety of ginseng, recognized as Asian or Korean ginseng, a perennial herb frequently grown in East Asia and prized for its roots, abundant in ginsenosides and various phytochemicals. In parallel, BPG refers to PG that has been transformed explicitly through enzymatic or microbial processes to change its primary ginsenosides (e.g., Rb1, Rc, Re) into less common minor ginsenosides like Compound K, Rg3, and Rh2, which show greatly improved bioavailability and enhanced antioxidative, anti-inflammatory, anticancer, and antidiabetic effects [29,30]. Additionally, bioconverted and BWG merge wild-simulated growth cultivating PG in forest-like settings for richer, more varied ginsenoside compositions with post-harvest bioconversion that increases rare, highly bioactive ginsenosides (like Compound K) and improves bioavailability, combining the phytochemical intricacy of natural development with the efficacy enhancements of biotransformation [31]. Nonetheless, low bioavailability and metabolic processing frequently limit its therapeutic effectiveness. BPG and BWG, enhanced variants of ginseng processed through enzymatic transformation, increase the accessibility of bioactive compounds, thereby boosting their therapeutic efficacy. Recent findings indicate that BWG has strong cytoprotective effects in various disease models [32,33]; however, its role in ischemic renal damage remains largely uninvestigated. Therefore, it was proposed that BPG and BWG, which Basidiomycota transform, would reduce oxidative renal damage and slow the advancement of renal I/R injury.

## 2 Materials and methods

### 2.1 Preparation of ginseng samples

PG extract (solid content 70 %) was provided by the Institute of Jinan Red Ginseng (Jinan-up, Jinan-gun, Jeonbuk, Korea). The WSG extract was prepared from whole plants cultivated for four years in the Bugui-myeon area of Jinan-gun (Jeonbuk, Korea). The plants were thoroughly washed, dried at 60 °C for 12 h, and pulverized. The powdered sample was extracted with 10 vol of 70 % ethanol using ultrasonic treatment for 30 min at room temperature, followed by centrifugation at 8000 rpm for 20 min. The residue was extracted twice more under the same conditions, and the three extracts were combined and concentrated under reduced pressure at 50 °C to yield the WSG extract (solid content 70 %). In addition, a root extract of WSG was prepared from roots with aerial parts removed, following the same procedure with 70 % ethanol, and adjusted to a solid content of 70 %.

### 2.2 Ginsenoside standards

Authentic standards (ginsenosides Rb1, Rb2, Rc, Rd, Rg3 (S), F2, Rh2 (S), and compound K (C-K) were kindly donated by the Korea Ginseng Corporation Research Institute (Daejeon, Republic of Korea). Authentic standards of C-Mc1, C-Mc, C-O, and C-Y were prepared from ginsenoside Rc or Rb2 using a crude enzyme preparation isolated from the mycelial mass of Armillaria mellea according to our previously reported methods [34,35].

### 2.3 Cultivation of *Ganoderma lucidum* (GL) seed culture

GL (known as Reishi or Lingzhi mushroom) belongs to the phylum Basidiomycota. GL (KACC 42231) was obtained from the Korean Agricultural Culture Collection (KACC, Jeonju, Jeonbuk, Republic of Korea). For seed culture preparation, 400 mL of pre-saccharified malt medium (11 °Brix) was transferred into a 2-L Erlenmeyer flask and inoculated with agar-grown mycelia. The culture was incubated at 25–26 °C on a rotary shaker at 120 rpm for 7 days to yield the liquid seed culture.

### 2.4 Preparation of fermented ginseng extract

Fully ripened fruits of *Maclura tricuspidata* (Carrière) Bureau were purchased from a farm in Milyang, Gyungnam, Republic of Korea, in November 2023. The fruits were blended with three volumes of water using a household mixer (Shinil Industrial Co., Ltd., Cheonan, Chungnam, Republic of Korea) for 30 s. The homogenate (200 mL, solid content 10 %) was combined with 10 g of ginseng extract (5 % w/w of the fruit mash) in a 500 mL Erlenmeyer flask and sterilized at 121 °C for 20 min. After cooling to room temperature, the samples were inoculated with 5 % (v/v) of pre-cultured liquid seed inoculum and incubated at room temperature for 15 days. Samples were collected periodically during incubation.

### 2.5 HPLC analysis

HPLC analysis was performed using an HPLC system (Waters, Millford, MA, USA) equipped with a 2690 separation module and 996 photodiode array detector with a YMC-Pack Pro C18 RS column (250 mm × 4.6 mm, 5 mm ID; YMC Co., Ltd, Shimogyo-ku, Kyoto, Japan). The mobile phase consisted of water (A) and acetonitrile (B) at ratios of (A: B) 70:30 (0–5 min), 70:30 (5–15 min), 43:57 (15–25 min), 30:70 (25–30 min), and 70:30 (30–40 min) at a flow rate of 0.8 mL/min. Ginsenosides were detected at 203 nm. The quantification of each ginsenoside was done using the external standard method using serially diluted standard mixtures (1.953–500 μg/mL).

### 2.6 Experimental animals

Six-week-old male C57BL/6 mice (Hanil Laboratory Animal Center, Wanju, South Korea) were acclimated for one week under standardized conditions (12 h light/dark cycle, 22 ± 2 °C, 50 % ± 5 % humidity) with unrestricted access to food and water. All procedures conformed to ethical guidelines and were approved by the Jeonbuk National University IACUC (Approval No. NON2024-086).

### 2.7 Experimental groups and treatment

The mice were assigned to this study after being randomly split into six blinded groups (n = 60 mice, 10 mice each). Due to its documented ability to prevent renal I/R injury, the antioxidant ascorbic acid was used as a positive control (PC) [36]. Following acclimatization, mice were randomized into six groups: (1) **Sham**—sham surgery with oral saline; (2) **I/R**—saline pretreatment orally with renal I/R induction; (3) **PC**—ascorbic acid (300 mg/kg) orally for 7 days before I/R; (4) **PG**—PG (25 mg/kg) pretreatment orally for 7 days before I/R; (5) **BPG**—BPG (25 mg/kg) orally for 7 days before I/R; and (6) **BWG**—BWG (25 mg/kg) orally for 7 days followed by I/R.

### 2.8 Induction of renal I/R and tissue harvest

A previously published protocol was followed to establish the bilateral I/R mouse model [37]. Male C57BL/6 mice (8 weeks, 20–25 g) were anesthetized with 1–2 % isoflurane and maintained at 37 °C using a heating pad and rectal probe. Renal ischemia was induced via dorsal application of microvascular clamps to bilateral renal pedicles for 30 min, with reperfusion confirmed by visible color change post-clamp release. Sham controls underwent identical procedures without clamping and were hydrated with warm saline. After 24 h of reperfusion, blood was collected, and bilateral nephrectomy was performed. Left kidneys were fixed in 4 % formaldehyde for histology; right kidneys were snap-frozen in liquid nitrogen and stored at −80 °C.

### 2.9 Histopathological evaluation

Paraffin-embedded kidney sections (5 μm) were stained with H&E to assess histopathological injury. Tubular damage—defined by brush border loss, ectasia, necrosis, hemorrhage, casts, and cell lysis—was graded on a 0–5 scale based on affected area: 0 (none), 1+ (<10 %), 2+ (10–25 %), 3+ (26–45 %), 4+ (46–75 %), 5+ (>75 %). Imaging was performed via microscope-mounted Opticam (KSS3-10 S; Sony, Japan) and analyzed using Optiview software (Korean Lab Tech).

### 2.10 Immunohistochemical analysis

Immunohistochemistry for TNF-α was conducted to assess inflammation in I/R-injured renal tissue. Sections (5 μm) from paraffin-embedded kidneys were incubated overnight at 40 °C, then heated to 60 °C for deparaffinization using xylene and rehydrated through graded ethanol. Endogenous peroxidase was blocked with 0.3 % H_2_O_2_–methanol. A commercial serum-free blocker was applied for 1 h, followed by TNF-α antibody (1:500) incubation at 4 °C overnight. Slides were washed, treated with a biotin-labeled secondary antibody (1:250) at 37 °C, then exposed to the ABC complex. After final washes, DAB chromogen developed the signal, counterstained with hematoxylin, and samples were mounted for microscopy.

### 2.11 TUNEL assay

To assess apoptosis in I/R-injured kidneys, TUNEL staining was performed per manufacturer protocol (HRP-DAB, ab206386). Paraffin sections were deparaffinized in xylene, rehydrated through ethanol, and washed with PBS. Samples were treated with Proteinase K, blocked with H_2_O_2_–methanol, and pre-equilibrated. The TdT reaction mix was applied, incubated, stopped, washed, and incubated with a conjugate. DAB-enabled chromogenic detection, followed by methyl green counterstain, mounting, and light microscopy.

### 2.12 Biochemical marker analysis

The blood collected in serum separator tubes (SST) was left at room temperature for 15 min, followed by centrifugation at 4500×*g*, 4 °C for 10 min. The supernatant harvested was used as serum. The collected serum was analyzed for BUN and Cr concentrations using Olympus AU2700 Plus (Olympus Corp., Tokyo, Japan) to assess renal function.

### 2.13 HK-2 cell culture

HK-2 cells (Korean Cell Line Bank, Seoul, South Korea) were cultured in DMEM (Gibco, Carlsbad, CA, USA) enriched with 10 % FBS and antibiotics. Incubation was conducted at 37 °C under 5 % CO_2_ using an SMA-30D incubator (ASTEC KOREA, Seoul). To control confluency and sustain viable proliferation, cells were dissociated every 72 h with 0.05 % trypsin and 0.02 % EDTA (WELGENE Inc., Gyeongsangbuk-do, South Korea).

### 2.14 Cell viability and cytotoxicity assay

Cell viability and cytotoxicity were assessed using MTT (Sigma-Aldrich, St. Louis, MO, USA). HK-2 cells were pretreated with PG, BPG, or BWG at varying doses for 4 h. To assess viability, cells were then co-incubated with 400 μM H_2_O_2_ (Fujifilm Wako, Osaka, Japan) for 24 h. Following treatment with 0.05 mg/mL MTT, cells were incubated for 2 h. Formazan crystals were solubilized in DMSO, and absorbance at 570 nm was measured using a Versa Max microplate reader (Molecular Devices, San Jose, CA, USA).

### 2.15 Malondialdehyde (MDA) assay

Lysis was performed using a RIPA/T-PER buffer system (Biosesang; Thermo Fisher) supplemented with protease (Roche, Cat#4693159001) and phosphatase (Sigma-Aldrich, Cat#4906845001) inhibitors. Cells and tissues were sonicated in the prepared buffer, and lysates were centrifuged at 12,000×*g* for 15 min at 4 °C. Supernatant protein content was quantified via BCA assay (Thermo Fisher), and malondialdehyde (MDA) levels were determined using a commercial kit (Cayman, Cat#10009055), expressed as nmol/mg protein.

### 2.16 Western blotting

Proteins were resolved via SDS-PAGE with equal sample loading, then transferred onto nitrocellulose membranes and blocked using 5 % BSA (Sigma-Aldrich). Primary antibodies (Supplementary Table 1) were diluted per manufacturer guidelines and incubated overnight at 4 °C. Membranes were subsequently treated with goat anti-rabbit IgG-HRP secondary antibody (sc2004; Santa Cruz) for 2 h. Bands were visualized using Clarity ECL substrate (Bio-Rad) and captured with Amersham™ ImageQuant™ 500 (Cytiva). Quantification was performed using ImageJ 1.50e.

### 2.17 Statistical analysis

Statistical analyses were performed using GraphPad Prism 8 (GraphPad Software Inc., La Jolla, CA, USA). Data are expressed as mean ± SD. Group comparisons were assessed via one-way ANOVA followed by Tukey's post hoc test. Significance was defined as *p* < 0.05.

## 3 Result

### 3.1 GL-mediated fermentation boosted minor ginsenosides (NGs) accumulation

When BPG (Supplementary Fig. 1A) or BWG (Supplementary Fig. 1B) was fermented via GL for 15 days, a high amount of NGs was produced between 5 and 15 days. At that time frame, HPLC fingerprinting revealed an overabundance peak for Mc, Rg3, CK, and Rh2 in the case of BWG, while Rg3 and CK were present in BPG.

### 3.2 PG, BPG, and BWG exhibited a non-cytotoxic effect on HK-2 cells

Cell viability was maintained above 95 % at all concentrations when HK-2 cells were pretreated with each extract at 2.5–25 μg/mL, and no statistically significant cytotoxicity was seen up to the 25 μg/mL concentration (Supplementary Fig. 2A–C). This implies that PG, BPG, and BWG are safe chemicals that do not affect kidney cells and are not hazardous to HK-2 cells.

### 3.3 BPG and BWG suppressed H_2_O_2_-induced in HK-2 cells

Treating HK-2 cells with H_2_O_2_ reduced their viability; at a concentration of 400 μM, their viability was 49.59 % (p < 0.05). The applied concentration of H_2_O_2_ was chosen at 400 μM in the following studies since it dropped to ≤30 % at higher concentrations (Supplementary Fig. 2D). A 400 μM treatment with H_2_O_2_ was subsequently used to generate oxidative stress after PG, BPG, and BWG had been pretreated at varying concentrations (5, 10, and 25 μg/mL) (Supplementary Fig. 2E). While PG did not significantly vary, BPG and BWG dramatically enhanced cell viability in a concentration-dependent manner by preventing H_2_O_2_-induced cell death. These findings suggest that while PG offers no protective benefits, BPG and BWG biotransformed by Basidiomycota efficiently reduce renal cell damageinduced by oxidative stress.

### 3.4 BPG and BWG escalated the expression of antioxidant enzymes

To determine the antioxidative effects of the extracts, we measured the expression of CAT, GPX-1, and SOD-1 enzymes using western blotting during both in vitro and in vivo phases. In contrast to the control/sham group, our findings indicated a notable reduction in enzymatic antioxidant activity in I/R mice (Fig. 1) and HK-2 cells (Fig. 2) treated with H_2_O_2_. However, there were notable concentration-dependent enhancements following pretreatment with BPG or BWG, particularly BWG, which exhibited similar effectiveness to the control group (p < 0.05; Fig. 2B–D) or sham group (p < 0.05; Fig. 1B–D). These data indicated that BWG (25 μg/mL) markedly restored CAT, GPX-1, and SOD-1 antioxidant enzymes in H_2_O_2_-challenged HK-2 cells, and BWG (25 mg/kg) exhibited renoprotection in I/K-AKT-induced mice via enhancing the antioxidant level.

**Fig. 1:**
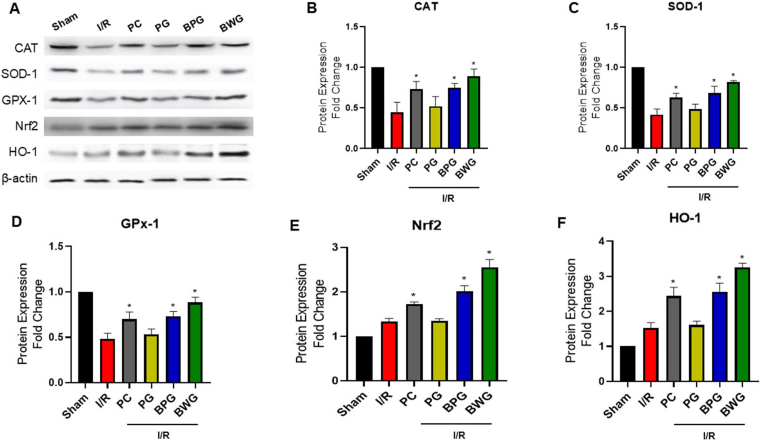
The antioxidative effect of extracts on renal injury caused by I/R. (A) Western blot analysis of kidney tissues from the Sham, I/R, PC, PG, BPG, and BWG groups. PG, BPG, and BWG extracts (25 mg/kg) were administered orally 7 days before I/R injury. (B–F) Quantification of (B) CAT, (C) SOD-1, (D) GPX-1, (E) Nrf2, and (F) HO-1 levels in kidney tissues collected 24 h after I/R injury. Protein expression levels were normalized to β-actin and presented as relative expression. Data are presented as the mean ± SD of triplicate experiments. Statistical analysis was performed using ANOVA followed by Tukey's multiple comparison test. ∗Indicates a significant difference compared with I/R (p < 0.05).

**Fig. 2:**
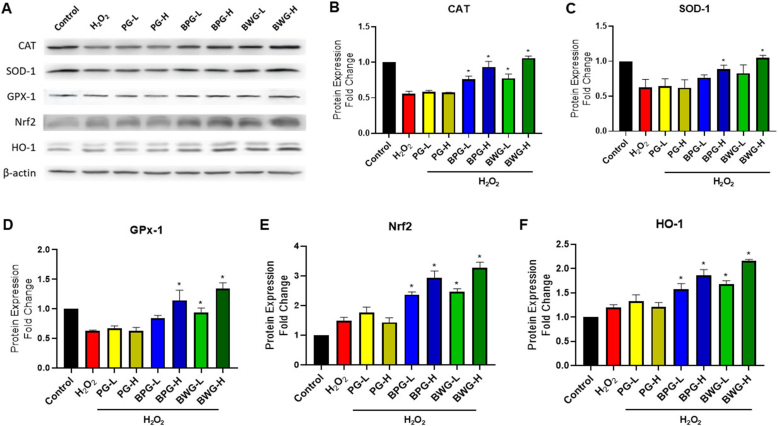
The antioxidative effect of extracts on cellular damage induced by H_2_O_2_. (A) Western blot analysis of HK-2 cells from the Control, H_2_O_2_, PG, BPG, and BWG groups. Cells were pretreated with low (L, 5 μg/mL) or high (H, 25 μg/mL) concentrations of PG, BPG, or BWG, followed by H_2_O_2_ treatment. (B–F) Quantification of (B) CAT, (C) SOD-1, (D) GPX-1, (E) Nrf2, and (F) HO-1 levels. Protein expression levels were normalized to β-actin and presented as relative expression. Data are presented as the mean ± SD of triplicate experiments. Statistical analysis was performed using ANOVA followed by Tukey's multiple comparison test. ∗Indicates a significant difference compared with H_2_O_2_ (p < 0.05).

### 3.5 BPG and BWG upregulated Nrf2/HO-1 expressions

The protective role of Nrf2/HO-1 signaling pathway activation in regulating antioxidant responses and cytoprotection has been proven in I/R models in previous studies [38]. BPW and BWG pretreatment displayed a notable upregulation of Nrf2 gene expression and subsequent elevation of HO-1 levels compared with the renal I/R group (P < 0.05) (Fig. 1E–F) and H_2_O_2_-induced HK-2 cell group (Fig. 2E–F) in western blotting. However, BWG pretreatment (μg/ml) in H_2_O_2_-induced HK-2 cells and 25 mg/kg in I/K-AKI-induced mice) highlighted the highest therapeutic effect.

### 3.6 BPG and BWG mitigated I/R-induced renal inflammation

**Fig. 3:**
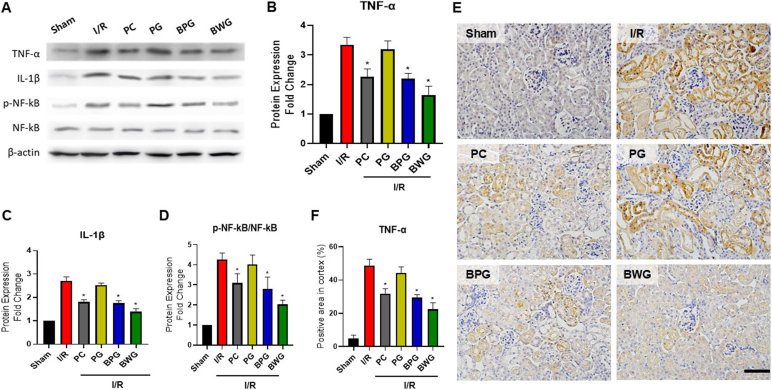
Protective effect of extracts on I/R-caused kidney injury through modulation of pro-inflammatory cytokines. (A) Western blot analysis of kidney tissues from the Sham, I/R, PC, PG, BPG, and BWG groups. PG, BPG, and BWG extracts (25 mg/kg) were administered orally 7 days before I/R injury. (B–D) Protein expression levels of (B) TNF-α, (C) IL-1β, and (D) NF-κB in kidney tissues were collected 24 h after I/R injury. Protein levels were normalized to β-actin and presented as relative expression. (E) Representative immunohistochemical (IHC) images of TNF-α expression in each group (Sham, I/R, PC, PG, BPG, and BWG) (Scale bar: 50 μm). (F) Quantification of TNF-α-positive cells per high-power field. Data are presented as the mean ± SD of triplicate experiments. Statistical analysis was performed using ANOVA followed by Tukey's multiple comparison test. ∗Indicates a significant difference compared with I/R (p < 0.05).

The expression of inflammation-related factors was analyzed to evaluate the anti-inflammatory effects of PG, BPG, and BWG in the I/R-AKI model. Compared with control, I/R induction increased the expression of inflammatory cytokines (TNF-α and IL-1β) and NF-κB phosphorylation. PG pretreatment showed no noticeable effect on regulating inflammatory factor expression, whereas BPG and BWG showed similar or significantly decreased expression to PC (p < 0.05; Fig. 3A–D). Moreover, TNF-α immunohistochemical results revealed a significant decrease in positive signal in BPG and BWG compared to that in I/R (p < 0.05; Fig. 3E and F). This suggests that biotransformed BPG and BWG can effectively inhibit I/R-induced inflammatory renal injury through their anti-inflammatory effects.

### 3.7 BPG and BWG alleviated apoptotic cell death

**Fig. 4:**
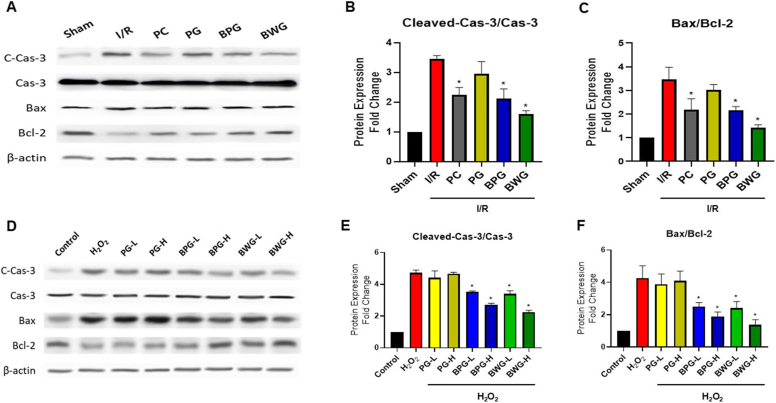
Extracts protect against kidney injury and HK-2 cell damage induced by I/R and H_2_O_2_ by modulating anti-apoptotic pathways. (A) Western blot analysis of kidney tissues from the Sham, I/R, PC, PG, BPG, and BWG groups. PG, BPG, and BWG extracts (25 mg/kg) were administered orally 7 days before I/R injury. (B, C) Quantification of (C) Bax/Bcl-2 ratio and (B) Cleaved-Caspase-3 ratio in kidney tissues collected 24 h after I/R injury. Protein levels were normalized to β-actin and presented as relative expression. (D) Western blot analysis of Control, H_2_O_2_, PG, BPG, and BWG groups. After pretreatment with low (L, 5 μg/mL) and high (H, 25 μg/mL) concentrations of PG, BPG, or BWG, HK-2 cells were exposed to H_2_O_2_, and the (F) Bax/Bcl-2 ratio and (E) Cleaved-Caspase-3 levels were evaluated. Protein levels are expressed as relative expression normalized to β-actin. Data are presented as the mean ± SD of triplicate experiments. Statistical analysis was performed using ANOVA followed by Tukey's multiple comparison test. ∗Indicates a significant difference compared with I/R or H_2_O_2_ (p < 0.05).

To further explore the renoprotective role of PG, BPG, and BWG, the protein expression of pro-apoptotic factor Bax, anti-apoptotic factor Bcl-2, and apoptotic enzyme Caspase-3 was examined by western blotting. The Bax/Bcl-2 and Cleaved-Caspase-3/Caspase-3 ratios were considerably increased (p < 0.05) increased in H_2_O_2_-treated HK-2 cells or I/R-induced mice (Fig. 4). Though no significant changes occur for PG pretreatment, pretreated BPG or BWG at the dose of 25 μg/mL (for H_2_O_2_-induced HK-2 cells) (Fig. 4E–F) and 25 mg/kg (for I/R-induced mice) (Fig. 4B–C) markedly (p < 0.05) reduce the Bax/Bcl-2 ratio and Cleaved-Caspase-3/Caspase-3 ratio. However, as compared with BPG, BWG had a greater regulation ability.

### 3.8 BPG or BWG pretreatment suppressed I/R-induced tubular epithelial cell apoptosis

**Fig. 5:**
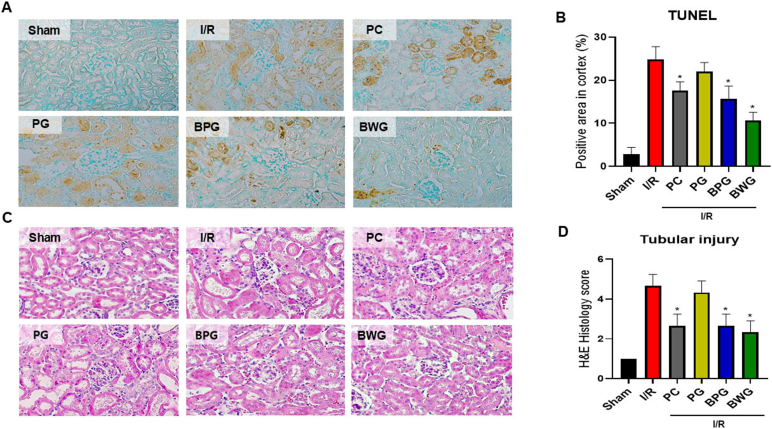
The administration of the extracts improves kidney injury caused by I/R, as assessed by the Tunnel assay. PG, BPG, and BWG extracts (25 mg/kg) were administered orally 7 days before I/R injury. Serum and kidney tissues were collected 24 h after I/R injury. (A) TUNEL staining was performed to detect apoptotic cells, with brown areas indicating positive regions. (B) Quantification of TUNEL-positive cells per high-power field. (C) Representative images of H&E-stained kidney sections from each group (Sham, I/R, PC, PG, BPG, and BWG) (Scale bar: 50 μm). (D) Quantitative analysis of tubular injury scores. Data are presented as the mean ± SD of triplicate experiments. Statistical analysis was performed using ANOVA followed by Tukey's multiple comparison test. ∗Indicates a significant difference compared with I/R (p < 0.05).

Additionally, the current study investigated the potential effects of PG, BPG, or BWG on the apoptosis of tubular epithelial cells. The sham group had a low number of apoptotic cells, according to the TUNEL assay, whereas I/R produced a markedly higher number of apoptotic cells, which were significantly decreased by pretreatment with BPG or BWG (Fig. 5A). These findings showed that BWG had the most excellent effectiveness (p < 0.05; Fig. 5B) in preventing tubular epithelial cells from I/R-AKI-induced apoptosis, despite PG's lack of discernible effectiveness.

### 3.9 BPG and BWG ameliorated histological damage after I/R

H&E staining was performed for histological analysis of I/R-AKI. Tubular damage and ectasia, luminal space collapse, cell necrosis, brush border loss, and interstitial neutrophil infiltration were characteristically identified in I/R and PG compared with the control (Fig. 5C). In contrast, some tubular damage and ectasia were observed in BPG and BWG. Still, the extent of damage was significantly reduced compared to I/R (p < 0.05; Fig. 5D), and they showed similar or better protective effects than ascorbic acid. These results suggest that BPG and BWG can effectively alleviate AKI functional decline induced by renal I/R.

### 3.10 BPG and BWG restrained MDA content generation

**Fig. 6:**
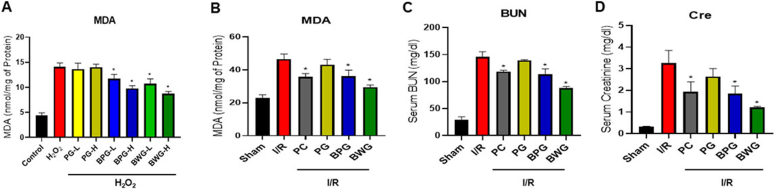
The administration of the extracts lowers H_2_O_2_-induced MDA levels in HK-2 cells, as well as I/R-induced MDA, BUN, and Cre levels. (A) MDA levels in HK-2 cells were pretreated with low (L, 5 μg/mL) or high (H, 25 μg/mL) concentrations of PG, BPG, or BWG, followed by H_2_O_2_ treatment. PG, BPG, and BWG extracts (25 mg/kg) were administered orally for 7 consecutive days prior to I/R injury. Kidney tissues were collected 24 h after I/R injury to measure (B) MDA, (C) BUN, and (D) Cre levels. Data are presented as the mean ± SD of triplicate experiments. Statistical analysis was performed using ANOVA followed by Tukey's multiple comparison test. ∗Indicates a significant difference compared with I/R or H_2_O_2_ (p < 0.05).

Exposure to H_2_O_2_ can lead to the formation of MDA, a marker of lipid peroxidation and oxidative stress. We thus assess the MDA assay to evaluate oxidative stress in H_2_O_2_ or I/R-AKI by measuring lipid peroxidation. Results showed a significant increase in MDA levels following H_2_O_2_ exposure (p < 0.05). However, pretreated BPG and BWG in HK-2 cells reduced MDA release in a concentration-dependent manner (p < 0.05; Fig. 6A). Similarly, I/R significantly increased the content of MDA, while the I/R-AKI mouse pretreated with BPG or BWG significantly reduced MDA levels (p < 0.05; Fig. 6B). Consequently, both extracts exhibit an antioxidative effect, with BWG displaying the most excellent efficacy.

### 3.11 BPG and BWG pretreatment ameliorated I/R-induced renal dysfunction

To understand the pathophysiological changes of PG, BPG, and BWG on renal I/R injury, AKI markers (serum BUN and Cr) and histological changes were analyzed. Biochemical analysis showed that I/R-induced AKI mice had dramatically increased levels of BUN and Cr compared to the control. Interestingly, PG pretreatment without biotransformation did not differ significantly from I/R. However, BUN and Cr changes were reduced considerably in BPG and BWG compared to I/R (p < 0.05; Fig. 6C and D).

## 4 Discussion

In this study, we investigated the protective effects of BPG and BWG on the kidneys and demonstrated their capacity to reduce renal I/R injury by modulating the Nrf2/HO-1/NF-κB/Caspase-3 signaling pathway.

Initially, we employed HPLC analysis to identify the biotransformed NGs. We found that BPG or BWG, when fermented for 15 days, produced a significant quantity of NGs from 5 to 15 days. During that timeframe, HPLC fingerprinting revealed a high peak for Mc, Rg3, CK, and Rh2 in BWG, whereas Rg3 and CK were noted in BPG. Our findings confirmed the presence of a previously unexamined compound in the sample, and its identity and properties have been validated using HPLC separation and detection.

Renal I/R injury remains a significant challenge in nephrology, primarily due to its complex pathophysiology, which encompasses oxidative stress, inflammation, and apoptosis. The initial phase of I/R injury is initiated mainly by radical reactions that occur rapidly upon reperfusion [39], where superoxide (O_2_−) is generated via the reverse electron transfer process [40]. Then, the O_2_ produced in mitochondria is converted into various ROS, including H_2_O_2_, hydroxyl radical (HO−), hypochlorous acid (HOCl−), nitric oxide (NO), and peroxynitrite (ONOO−) [41]. Among these, H_2_O_2_, an intermediate degradation product of ROS, is considered a significant trigger of oxidative damage due to its high reactivity [42]. It induces oxidative stress in vitro, including in the kidney proximal human tubule HK-2 cell line [43]. This is the first study to reveal that BPG and BWG can alleviate oxidative stress and may have the potential as preventive and therapeutic agents for ischemic renal injury.

Lipid peroxidation is represented by MDA, a standard biomarker of oxidative stress, and its level is continually evaluated to determine the degree of oxidative stress [44]. Endogenous antioxidants are responsible for scavenging free radicals and neutralizing oxidants, consisting of main enzymes such as CAT, GPX, and SOD [42,45]. SOD, the primary intracellular defense system, breaks down O_2_− into H_2_O_2_ and molecular oxygen. However, the accumulation of H_2_O_2_ is toxic to tissues and cells, so to prevent this, CAT in the peroxisome breaks down H_2_O_2_ into water and oxygen. GPX converts H_2_O_2_ to water in mitochondria and reduces lipid peroxides to alcohol, protecting organisms from oxidative damage [46]. Interestingly, compared to PG, BPG, and BWG effectively inhibited MDA production by reinforcing endogenous antioxidant expression of CAT, SOD-1, and GPX-1 in H_2_O_2_-induced HK-2 cells and the I/R-AKI mouse model. This suggests that BPG and BWG have potential as promising antioxidants that can alleviate ischemic renal injury. Previous studies revealed that ginsenoside-Rd successfully preserves the antioxidant marker levels in serum [47,48].

It is well established that Nrf2 is a transcription factor that functions upstream of the antioxidant defense system and is expressed in various cell types [49]. An enzyme called HO-1 is highly expressed when oxidative stress is triggered. It catalyzes the breakdown of heme and releases free iron, producing heme metabolites that have anti-inflammatory and antioxidant qualities [50]. The Nrf2/HO-1 signaling pathway maintains cellular homeostasis through oxidative stress regulation and antioxidant gene expression in ischemic kidney injury [51]. Consistent with previous studies [52], expression of Nrf2/HO-1 was increased in oxidative stress-induced HK-2 cells and I/R-AKI models due to pretreatment with BPG and BWG.

Inflammation serves as a critical aspect of renal I/R injury, evidenced by the infiltration of leukocytes, the upregulation of chemotactic agents by endothelial cells, and the production of pro-inflammatory mediators by renal tubular epithelial cells [53]. Pro-inflammatory cytokines TNF-α and IL-1β promote ROS amplification and activation of the NF-κB complex [54]. Additionally, the nuclear transcription factor NF-κB influences AKI severity, which is also involved in various physiological processes, including immune response, inflammation, and cell growth and survival [55]. According to our study, oral administration of BPG and BWG biotransformed by Basidiomycota reduced the levels of inflammatory cytokines and NF-κB expression induced by I/R, thereby mitigating inflammatory responses. BPG and BWG can exhibit potential anti-inflammatory effects in renal I/R injury by decreasing the interaction between ROS and the inflammatory factor.

Renal I/R induces severe oxidative stress and inflammatory damage, while the damaged cells are eliminated through pathophysiological apoptotic mechanisms [56]. Overexpression of the pro-apoptotic protein Bax or underexpression of the anti-apoptotic protein Bcl-2 creates pores in the mitochondrial outer membrane, leading to the release of cytochrome *c* into the cytoplasm [57]. The released cytochrome *c* activates caspase-3, converting it to its cleaved form, which cleaves various cellular proteins, ultimately leading to a typical apoptotic phenotype [58]. In this study, pretreatment with BPG and BWG decreased the Bax/Bcl-2 ratio and Cleaved-Caspase-3 expression in oxidative stress-induced HK-2 cells. The renal I/R injury model and the morphological anti-apoptotic effect were confirmed by TUNEL assay. These findings suggest that BPG and BWG can inhibit oxidative stress-induced renal cell death and may improve the prognosis of patients with renal I/R injury by regulating specific cell death pathways.

BUN and Cr are traditional and critical markers of renal function [59]. Most studies show decreased glomerular filtration rate with elevated serum BUN and Cr levels in I/R-induced AKI [60]. Consistent with previous studies, serum BUN and Cr levels increased in I/R, and necrosis was observed by H&E staining, demonstrating a validated model of renal I/R injury. However, oral administration of BPG and BWG alleviated serum BUN and Cr levels as well as histopathological tubular damage induced by I/R. These results suggest that BPG and BWG may help alleviate renal I/R injury.

In conclusion, experimental results indicated that the elevated levels of NGs identified in BPG and BWG were closely associated with their significant renoprotective effects against renal I/R injury, achieved through the reduction of oxidative stress, inflammation, and apoptosis. Our findings show that BPG and BWG activate the Nrf2/HO-1 pathway, thereby enhancing antioxidant defense and regulating oxidative enzymes to effectively reduce lipid peroxidation. At the same time, extracts block NF-κB signaling, reducing pro-inflammatory cytokine levels and lowering kidney inflammation. Additionally, extracts modulate caspase-3 activity, preventing apoptotic cell death and preserving renal tubular integrity. These protective effects enhance kidney function and lessen tissue damage after ischemia-reperfusion injury. Due to its ability to modulate multiple cellular pathways involved in oxidative stress, inflammation, and apoptosis, BWG shows potential as a therapeutic agent for the prevention and treatment of renal I/R injury.

## Author contributions

**Conception and design**: Byung-Yong Park and Ye Ji Kim; **Methodology**: Ye Ji Kim, Joonseok Lee and Md Shiblee Sadik Sabuj; **Software**: Ye Ji Kim; **Original draft preparation**: Ye Ji Kim, Md Shiblee Sadik Sabuj and Md Rashedunnabi Akanda; **Review and editing**: Byung-Yong Park, Ye Ji Kim, Md Rashedunnabi Akanda, Md Shiblee Sadik Sabuj, Joonseok Lee, Ryunhee Kim, Seung Hyun Lee, Jin Min Oh, Hyeon Gyeong Ro, In-Shik Kim, Dongchoon Ahn, Myung-Kon Kim, and Hyun-Jin Tae; **Supervision**: Byung-Yong Park and Hyun-Jin Tae. All authors read and approved the final version of the manuscript.

## Ethics approval and consent to participate

The study protocols were carried out following relevant guidelines and regulations approved by the animal welfare regulations of the Institutional Animal Care and Use Committee (approval no CBNU-2020-003) of the Jeonbuk National University Laboratory Animal Center in South Korea and in compliance with the ARRIVE guidelines.

## Availability of data and materials

All data are available from the corresponding author upon reasonable request.

## Consent for publication

Not Applicable.

## Generative AI statement

The authors declare that no Gen AI was used in the creation of this manuscript.

## Funding

This research was supported by the 10.13039/501100003725National Research Foundation (NRF) of Korea, grants funded by the Korean government (MIST) (RS-2023-00247813).

## Conflict of interest statement

The authors whose names are listed immediately below certify that they have NO affiliations with or involvement in any organization or entity with any financial interest (such as honoraria; educational grants; participation in speakers’ bureaus; membership, employment, consultancies, stock ownership, or other equity interest; and expert testimony or patent-licensing arrangements), or nonfinancial interest (such as personal or professional relationships, affiliations, knowledge or beliefs) in the subject matter or materials discussed in this manuscript.

## Figures and Tables

**figs1:**
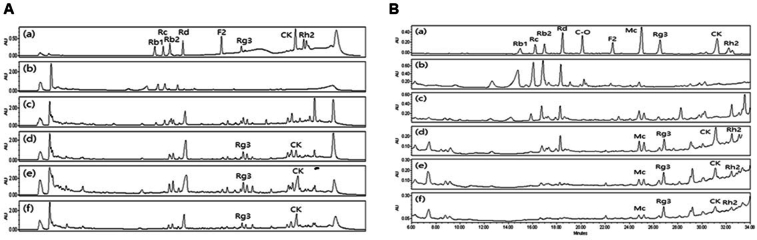


**figs2:**