# Supplementary material for: Renoprotective mechanisms of bioconverted wild-simulated ginseng: mitigating oxidative stress, inflammation, and apoptosis to protect against ischemic renal injury via Nrf2/HO-1/NF-κB/caspase-3 signaling
Source: J Ginseng Res. 2025 Oct 26;50(1):100910. doi: 10.1016/j.jgr.2025.10.007 (PMC12805545; doi:10.1016/j.jgr.2025.10.007)
Supplement: Multimedia component 1 [file mmc1.docx]

Table 1. Primary antibodies

| Antibody | Host | Manufacturer | Cat. No. | Dilution |
| --- | --- | --- | --- | --- |
| GPX 1 | Rabbit | Abcam, UK | 25301 | 1:1000 |
| β-actin | Rabbit | Cell Signaling, USA | 4970 | 1:5000 |
| BAX | Rabbit | Cell Signaling, USA | 5023 | 1:500 |
| Bcl-2 | Rabbit | Santa Cruz Biotechnology | sc-493 | 1:1000 |
| Caspase-3 | Rabbit | Cell Signaling, USA | 14220 | 1:1000 |
| C-Caspase-3 | Rabbit | Cell Signaling, USA | 9661 | 1:500 |
| HO-1 | Rabbit | Abcam, UK | Ab13243 | 1:2000 |
| IL-1 beta | Rabbit | Abcam, UK | Ab9722 | 0.1μg/ml |
| NF-kappa-B | Rabbit | Cell Signaling, USA | 8242 | 1:1000 |
| p-NF-kappa-B | Rabbit | Cell Signaling, USA | 3033 | 1:1000 |
| Nrf-2 | Rabbit | Novus Bio-tech, USA | NBP1-32822 | 1:1000 |
| SOD-1 | Rabbit | Abcam, UK | Ab13498 | 0.2μg/ml |
| TNF-α | Rabbit | Genetex, Canada | GTX110520 | 1:1000 |
| CAT | Rabbit | Abcam, UK | Ab16731 | 1:1000 |

Note: β-actin, beta-actin; GPX 1, glutathione peroxidase 1; SOD 1, superoxide dismutase 1; BCL-2, B-cell lymphoma 2 protein; BAX, BCL-2 associated X protein; Caspase-3, cysteine-aspartic acid protease 3; C-Caspase-3, cleaved Caspase-3; HO-1, Heme oxygenase-1; IL-1 beta, interleukin-1 beta; NF-kappa-B, nuclear factor kappa B; p-NF-kappa-B, phosphorylated form of NF-kappa-B; Nrf-2, nuclear factor erythroid 2-related factor-2; TNF-α, tumor necrosis factor alpha; CAT; catalase enzyme; Cat NO, catalog number.
