# Supplementary material for: Renoprotective mechanisms of bioconverted wild-simulated ginseng: mitigating oxidative stress, inflammation, and apoptosis to protect against ischemic renal injury via Nrf2/HO-1/NF-κB/caspase-3 signaling
Source: J Ginseng Res. 2025 Oct 26;50(1):100910. doi: 10.1016/j.jgr.2025.10.007 (PMC12805545; doi:10.1016/j.jgr.2025.10.007)

Supplementary Figure 1: The Uncropped immune blot data of Nrf2 in I/R-induced kidney (Figure 3). The red arrow indicates the location of the target bands

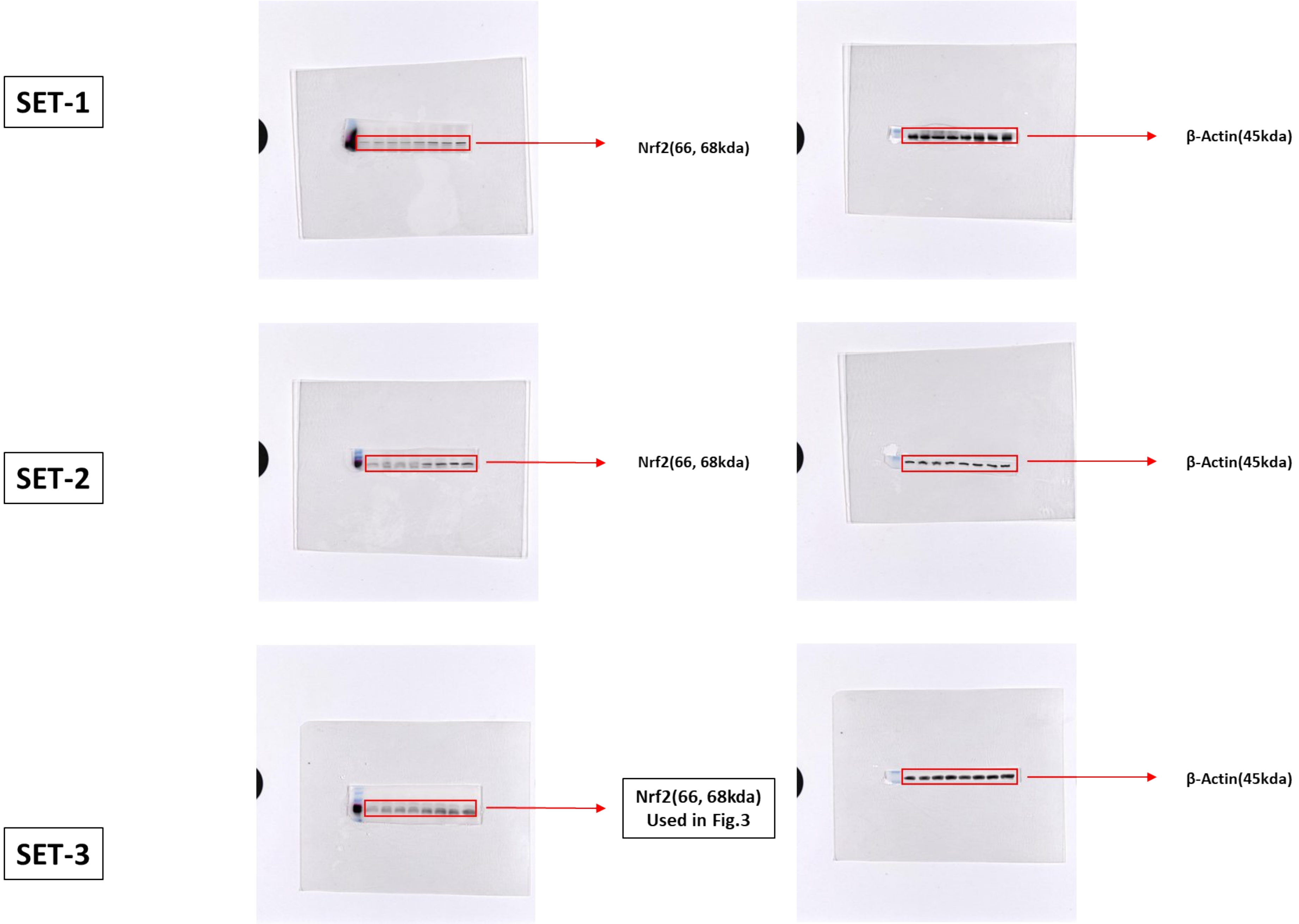

Supplementary Figure 2: The Uncropped immune blot data of HO-1 in I/R-induced kidney (Figure 3). The red arrow indicates the location of the target bands

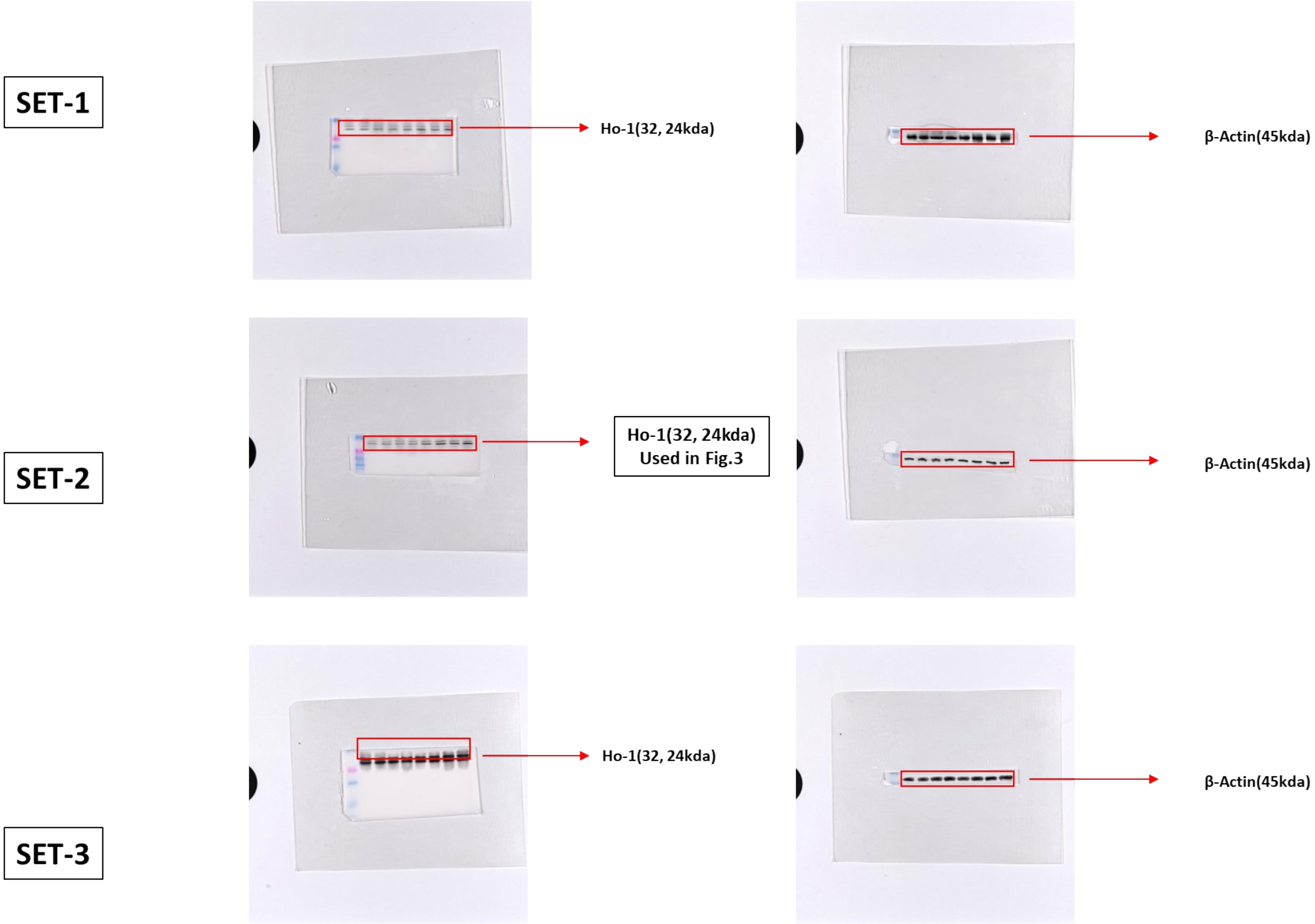

Supplementary Figure 3: The Uncropped immune blot data of CAT in I/R-induced kidney (Figure 3). The red arrow indicates the location of the target bands

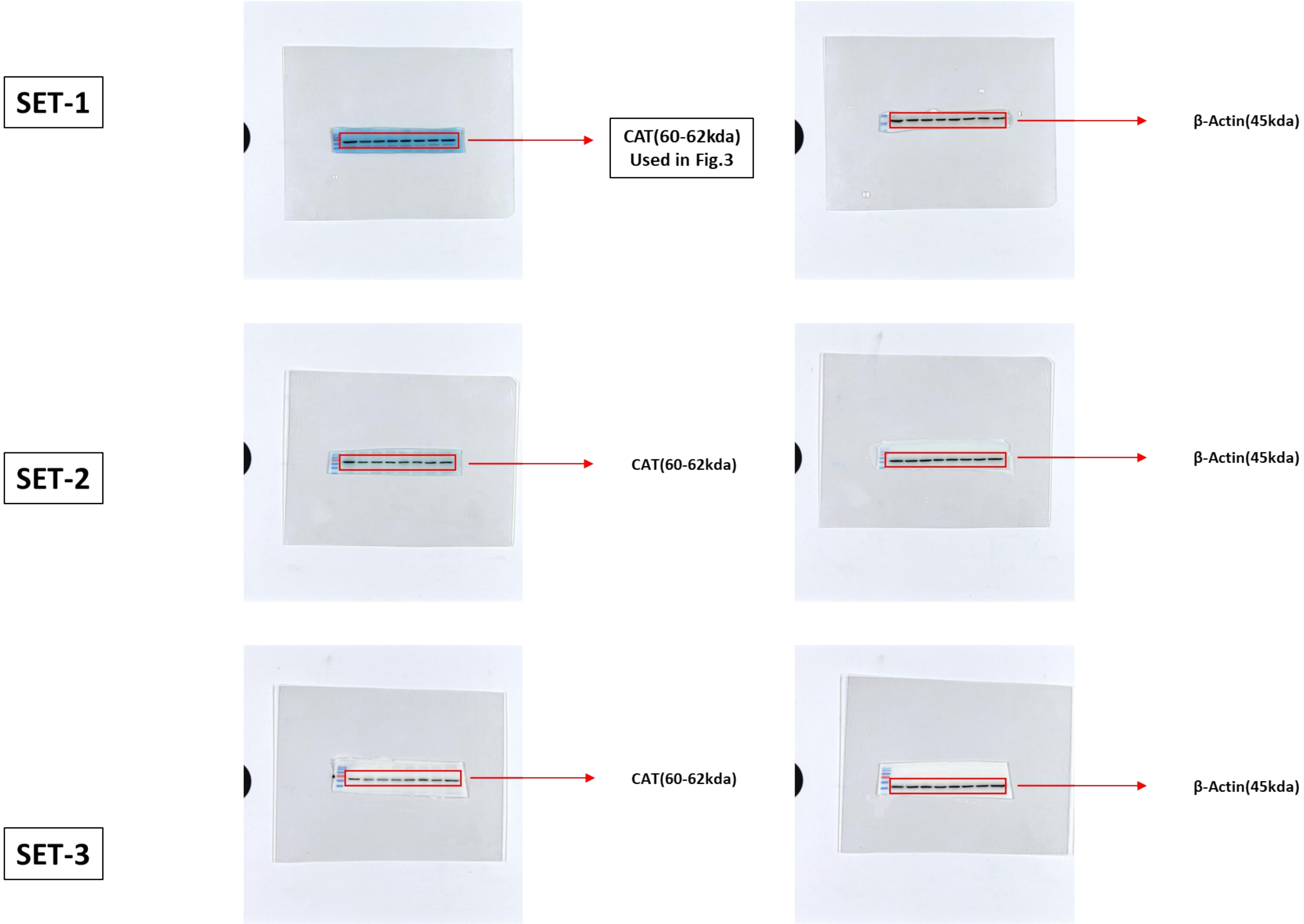

Supplementary Figure 4: The Uncropped immune blot data of GPX-1 in I/R-induced kidney (Figure 3). The red arrow indicates the location of the target bands

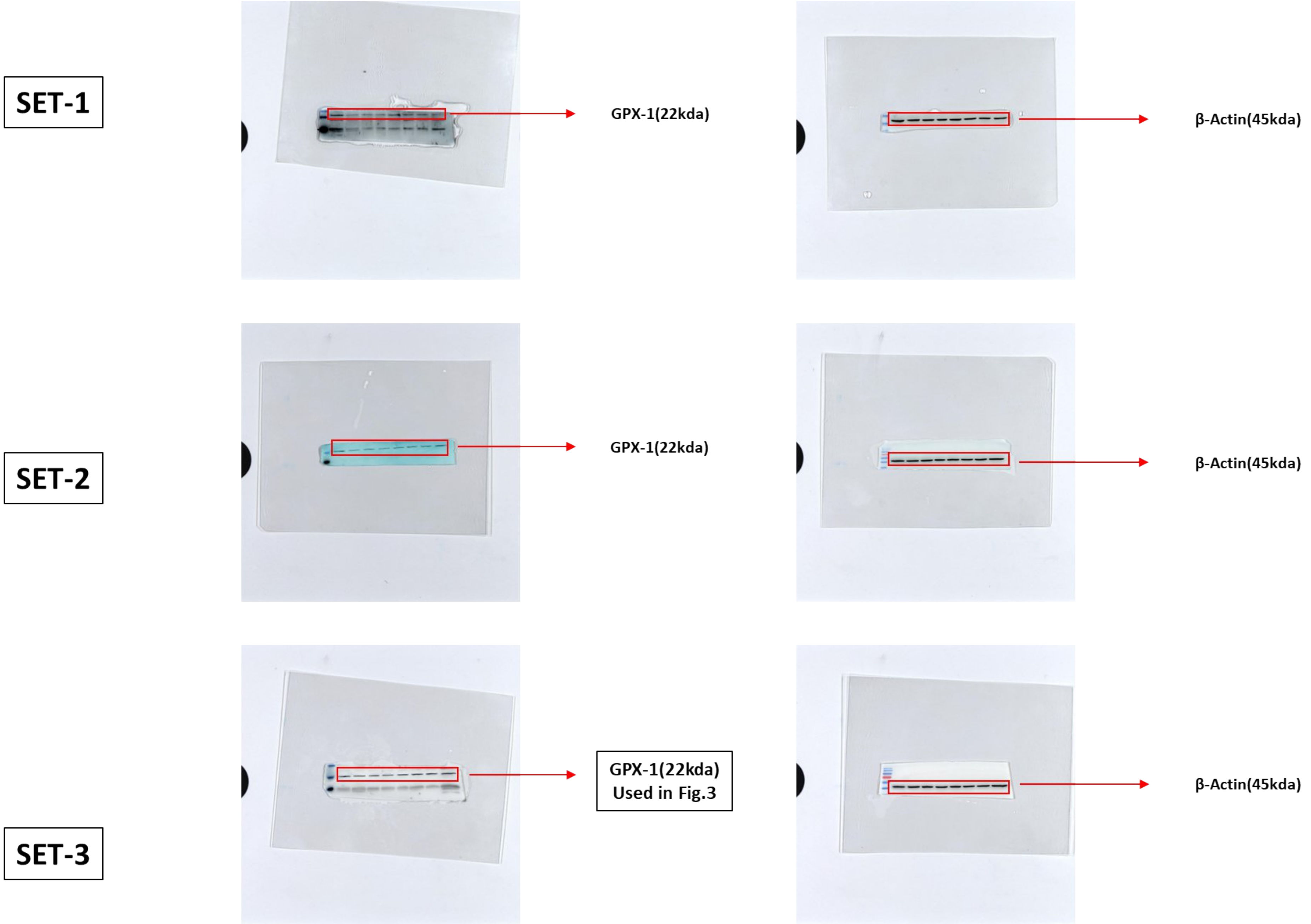

Supplementary Figure 5: The Uncropped immune blot data of SOD-1 in I/R-induced kidney (Figure 3). The red arrow indicates the location of the target bands

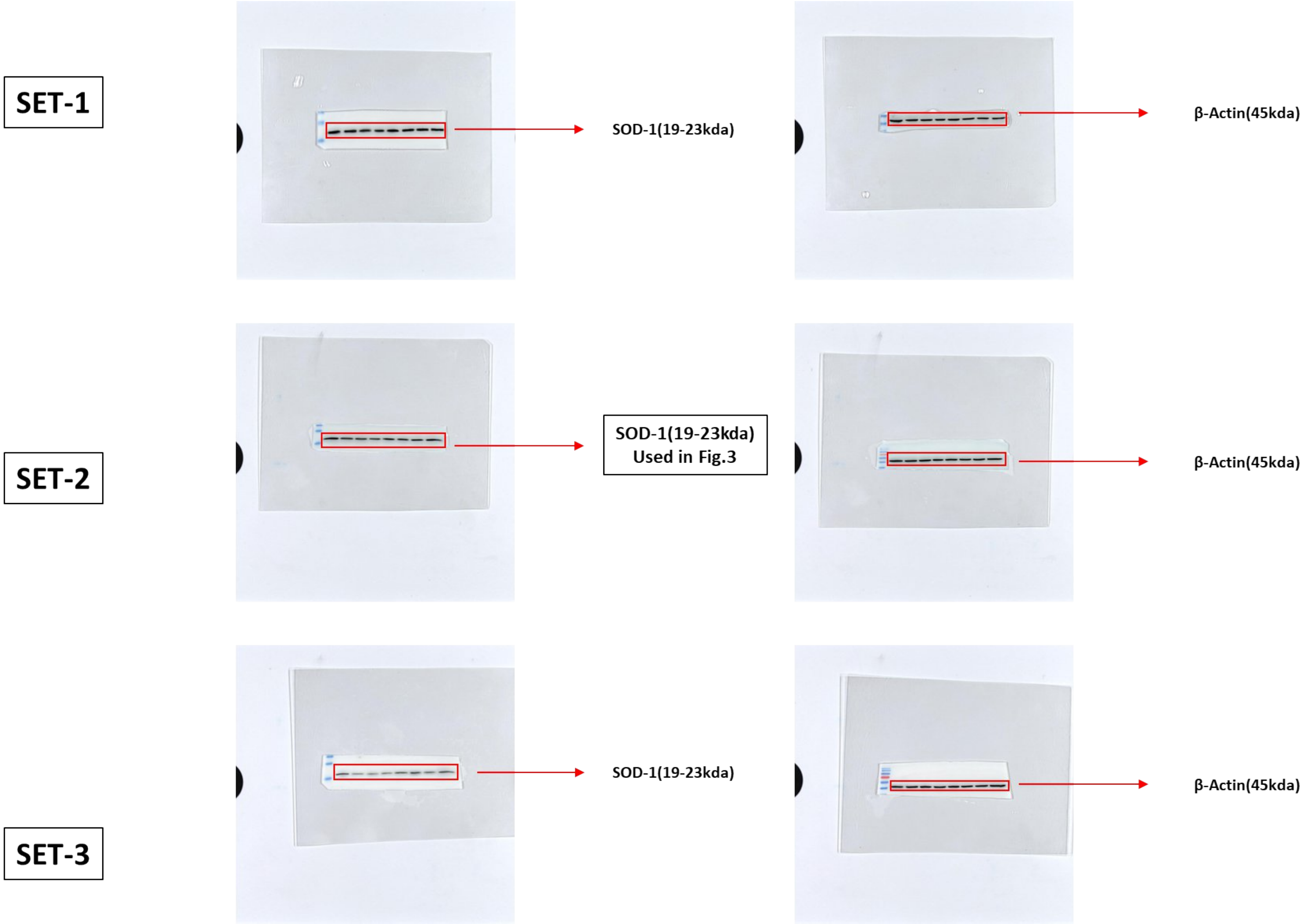

Supplementary Figure 6: The Uncropped immune blot data of Bax in H2O2-induced kidney (Figure 6). The red arrow indicates the location of the target bands

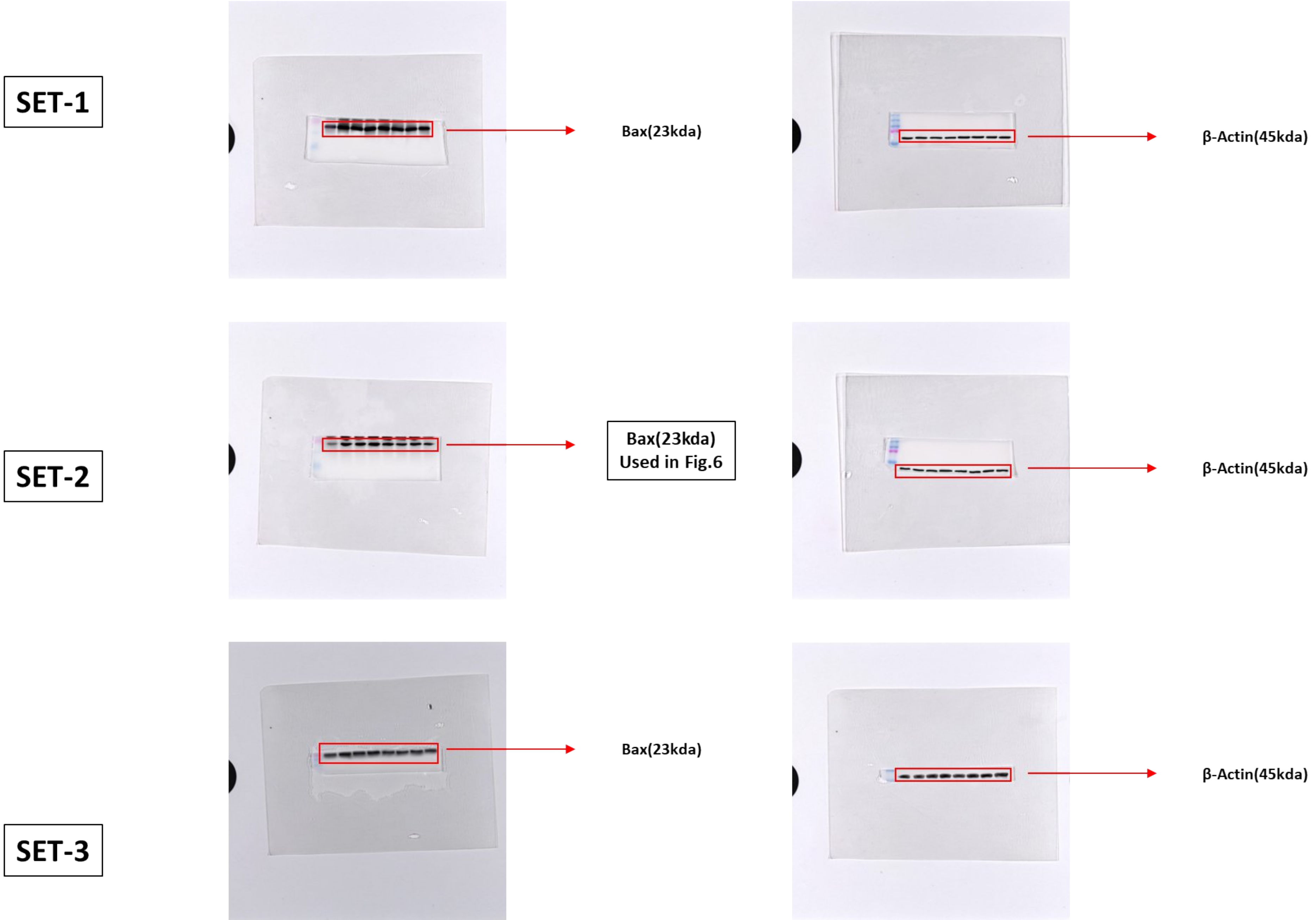

Supplementary Figure 7: The Uncropped immune blot data of Bcl-2 in H2O2-induced kidney (Figure 6). The red arrow indicates the location of the target bands

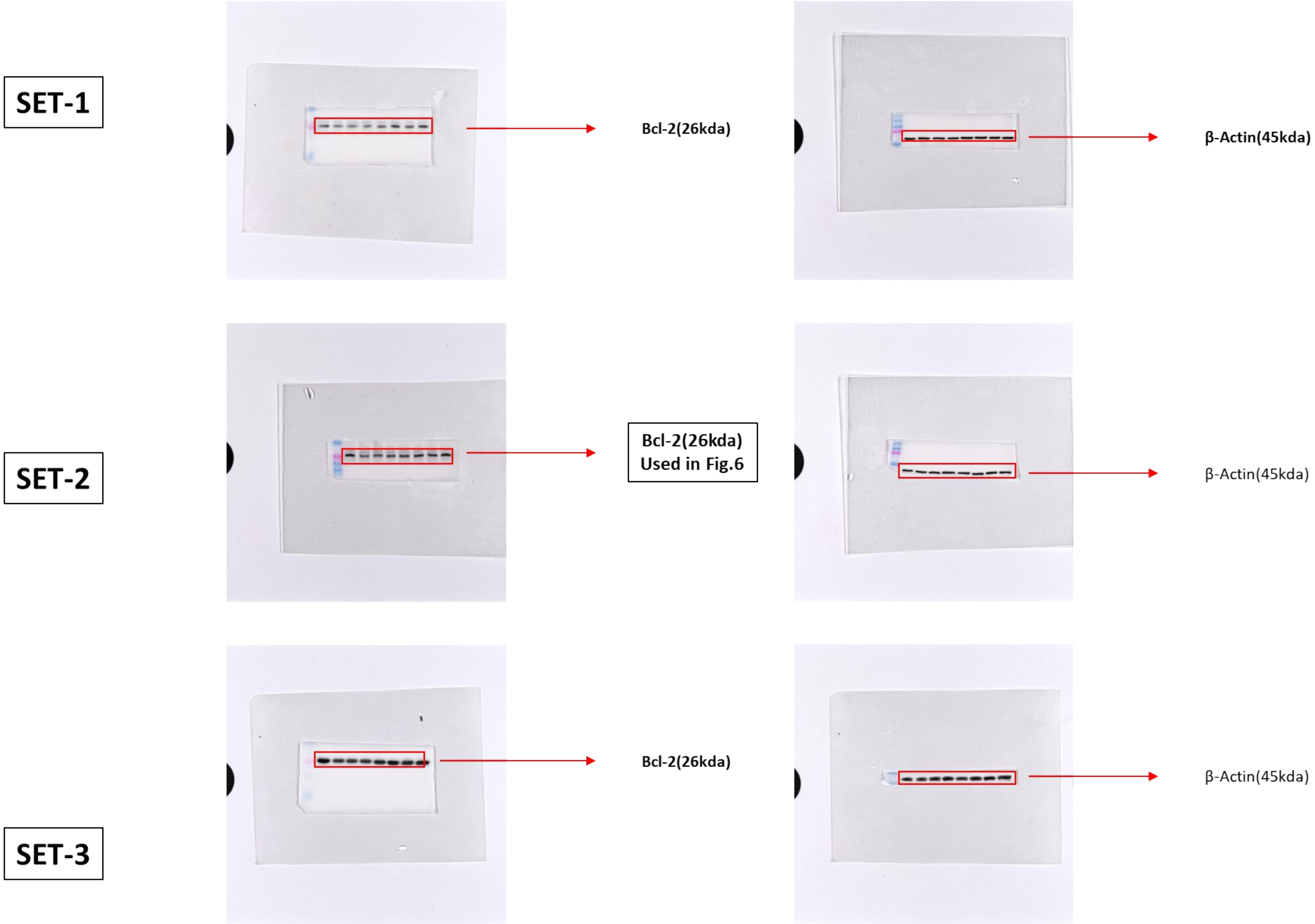

Supplementary Figure 8: The Uncropped immune blot data of C-Cas-3 and Cas-3 in H2O2-induced kidney (Figure 6). The red arrow indicates the location of the target bands

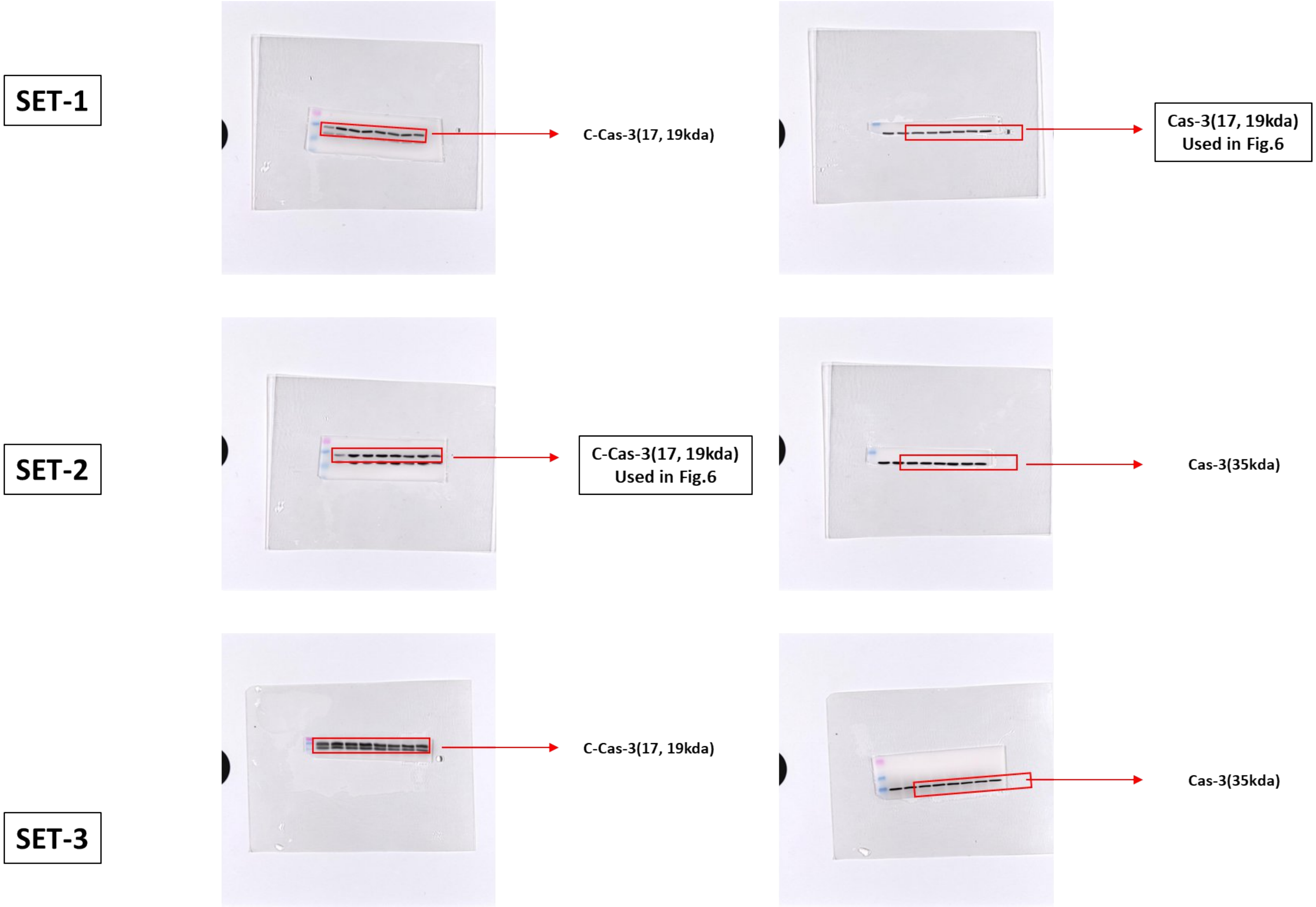

Supplementary Figure 9: The Uncropped immune blot data of Nrf2 in H2O2-induced kidney (Figure 4). The red arrow indicates the location of the target bands

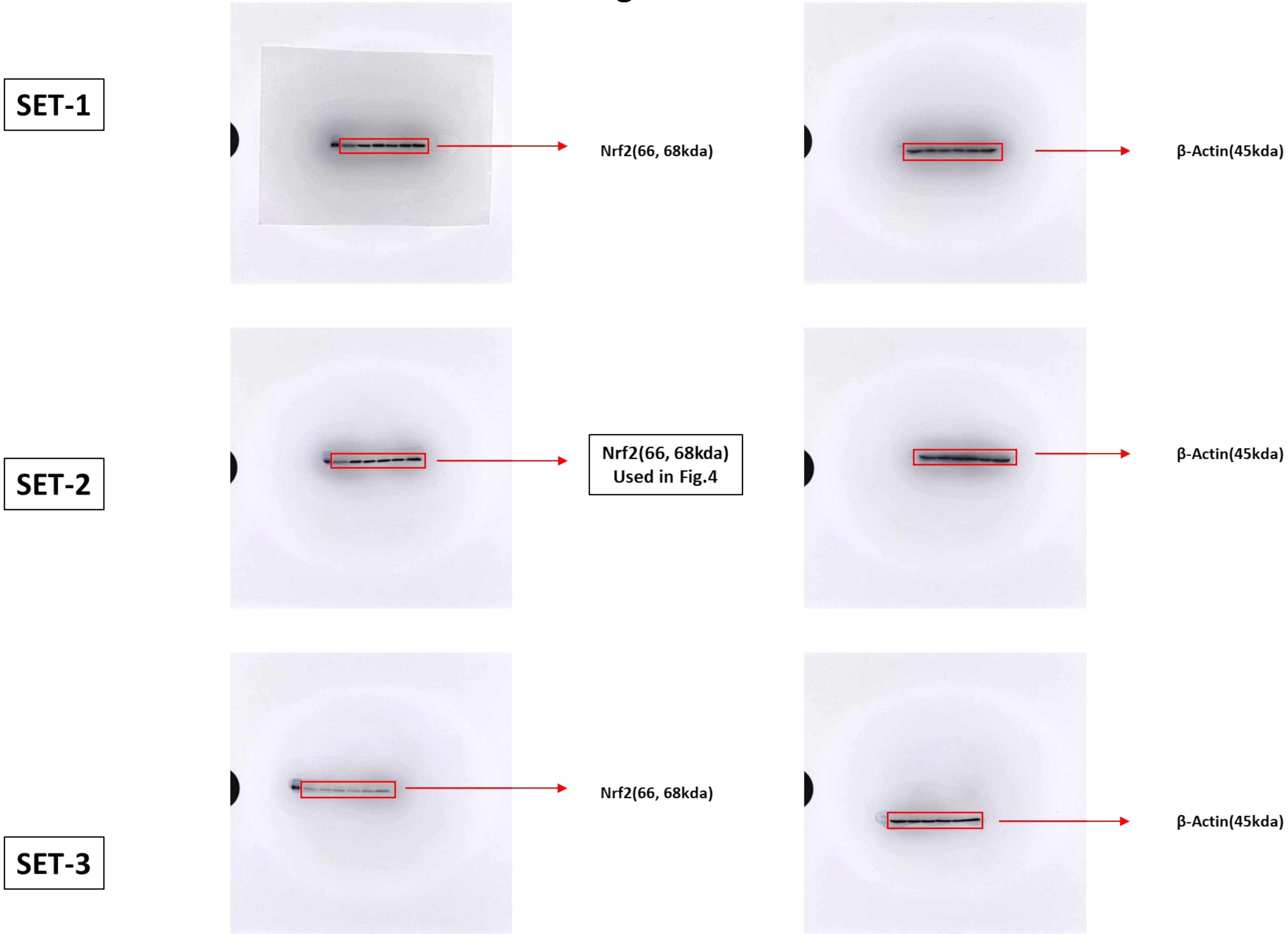

Supplementary Figure 10: The Uncropped immune blot data of HO-1 in H2O2-induced kidney (Figure 4). The red arrow indicates the location of the target bands

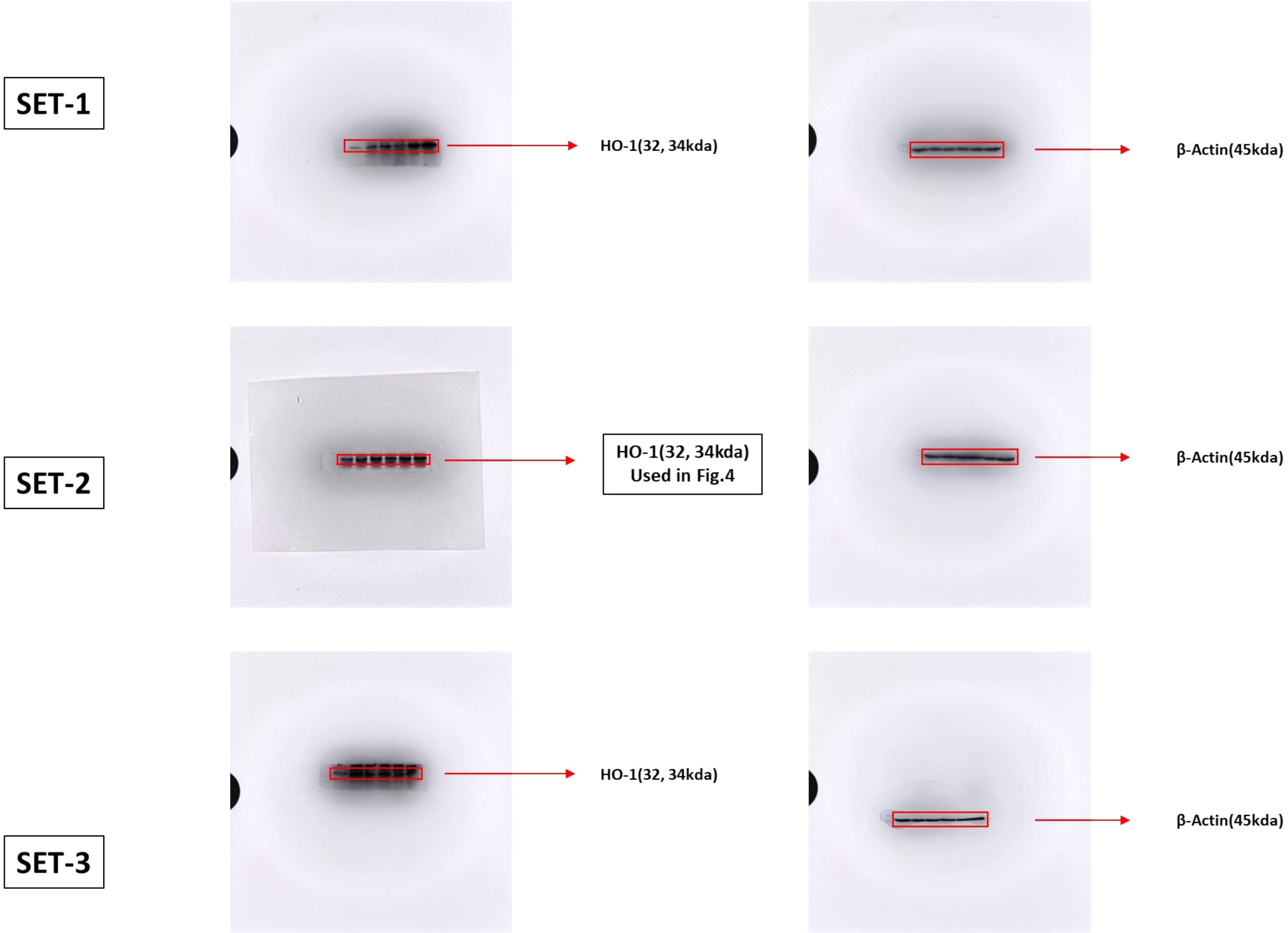

Supplementary Figure 11: The Uncropped immune blot data of CAT in H2O2-induced kidney (Figure 4). The red arrow indicates the location of the target bands

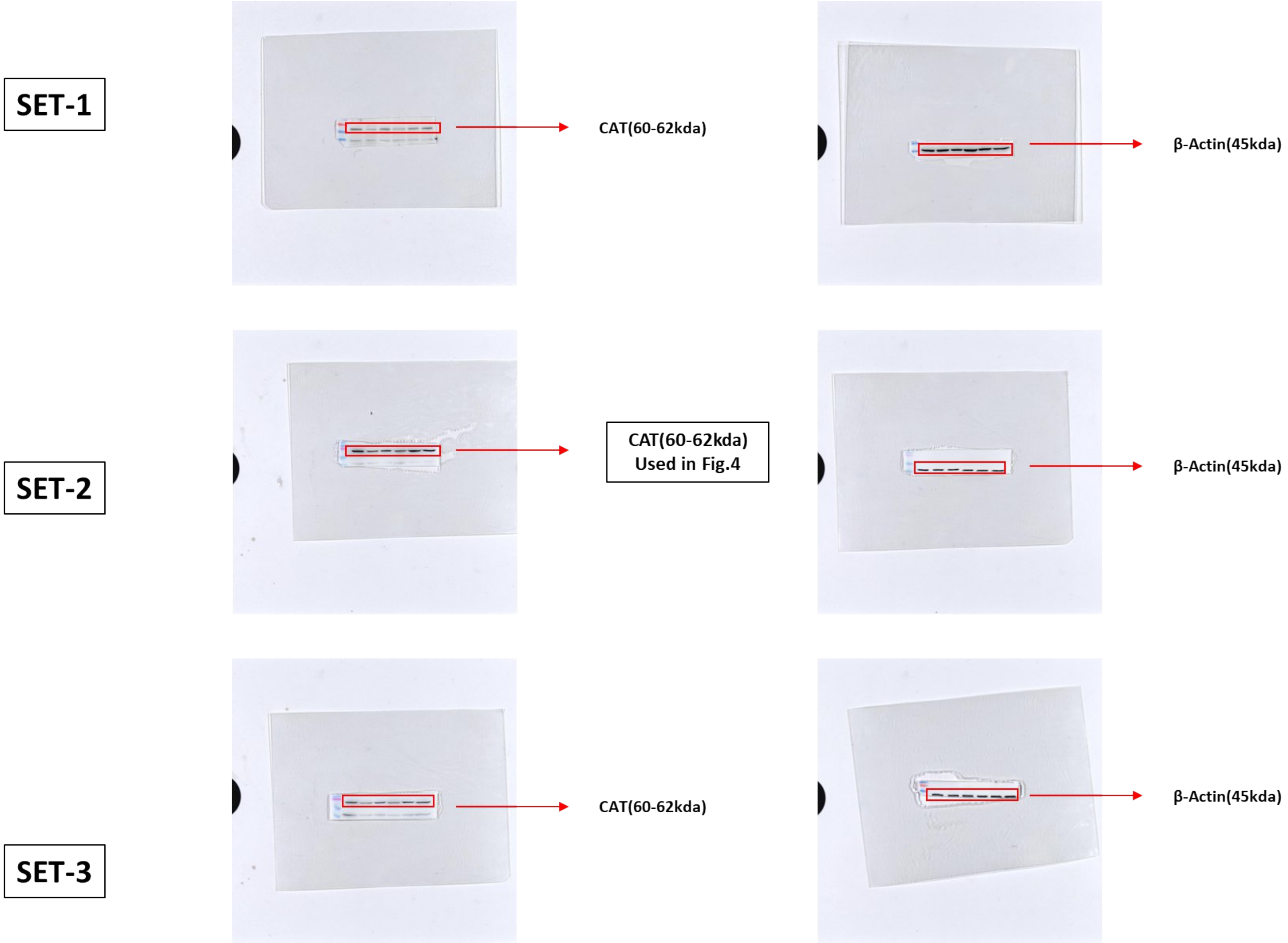

Supplementary Figure 12: The Uncropped immune blot data of GPX-1 in H2O2-induced kidney (Figure 4). The red arrow indicates the location of the target bands

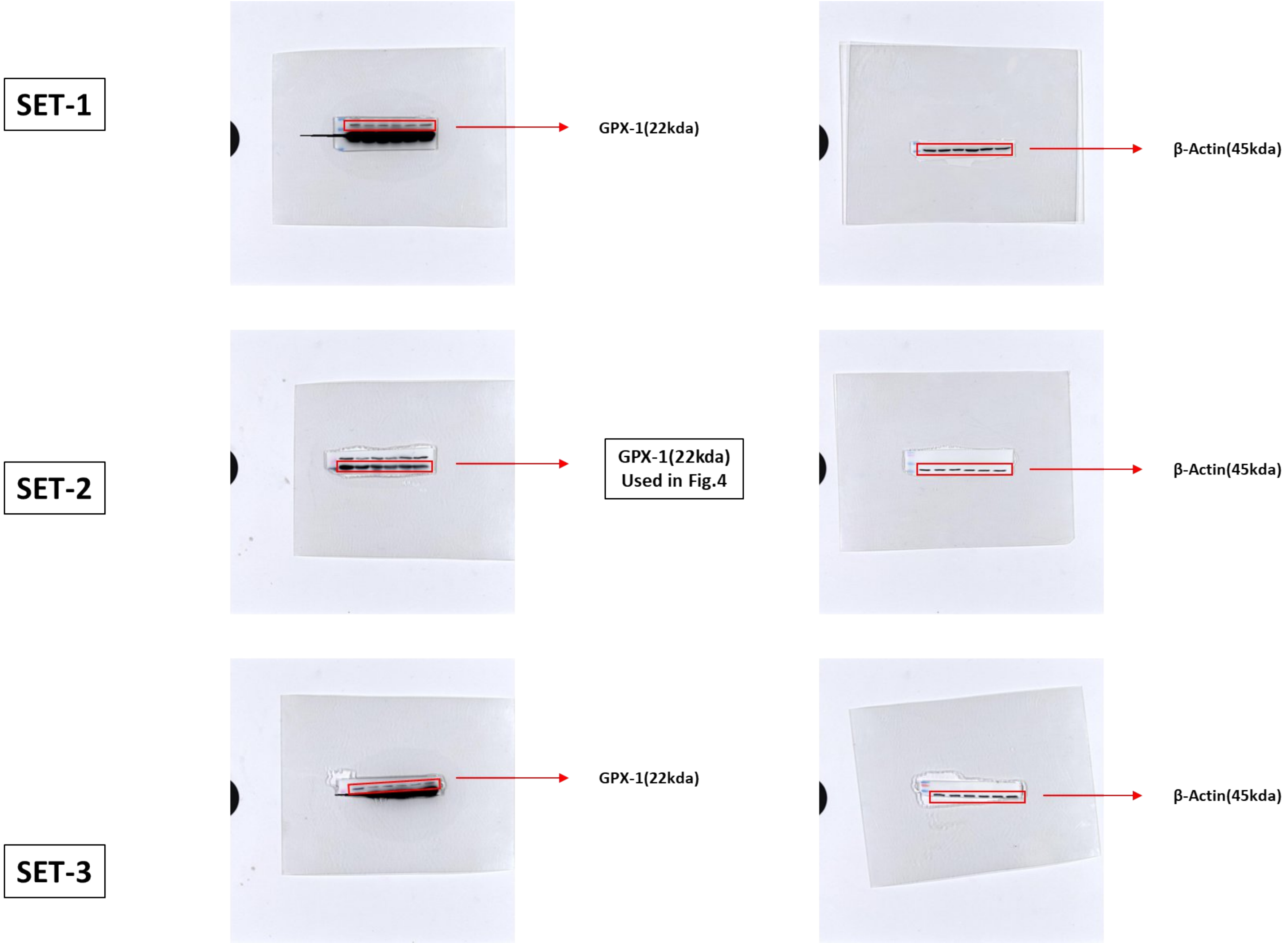

Supplementary Figure 13: The Uncropped immune blot data of SOD-1 in H2O2-induced kidney (Figure 4). The red arrow indicates the location of the target bands

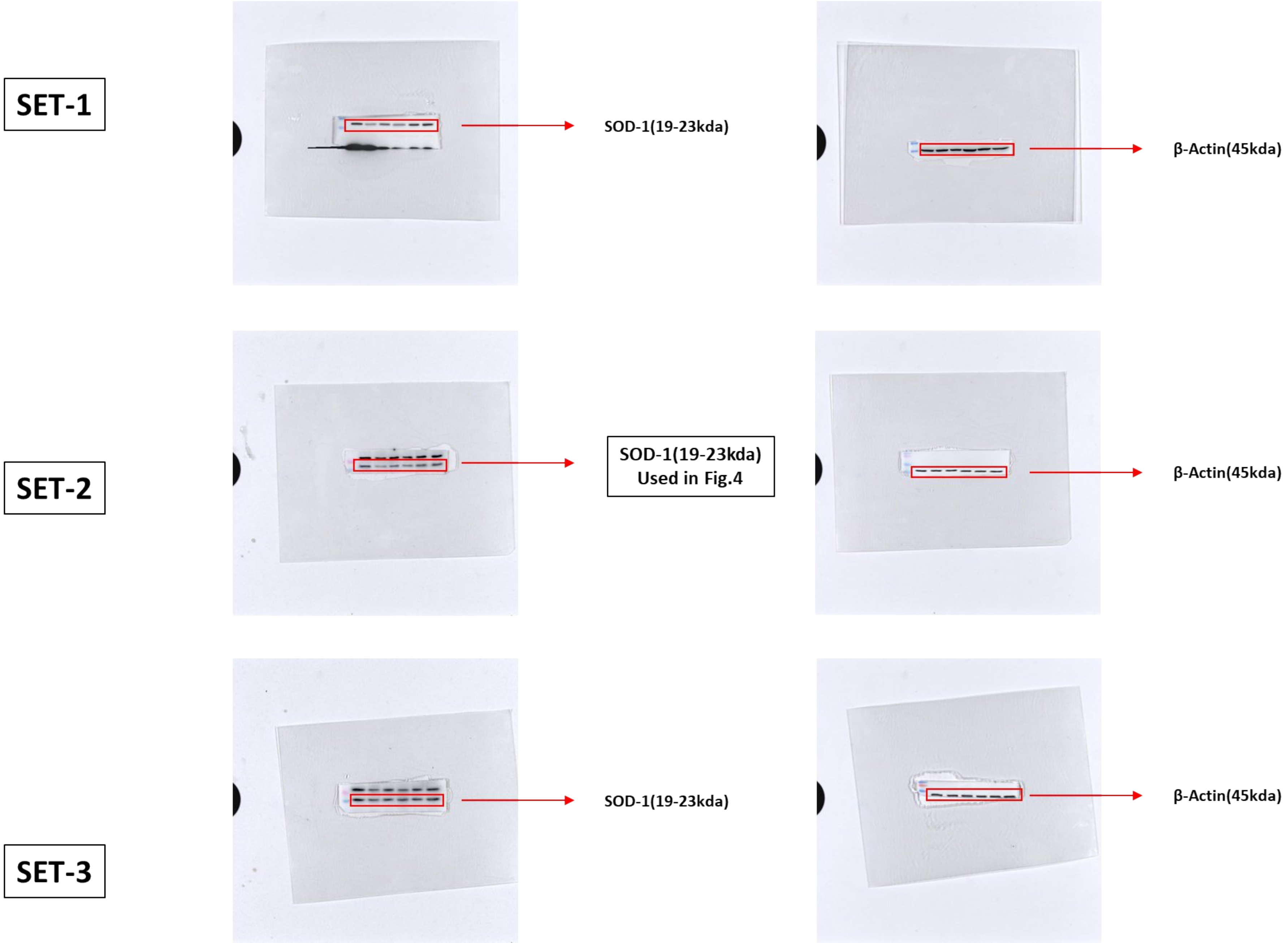

Supplementary Figure 14: The Uncropped immune blot data of Bax in I/R-induced kidney (Figure 6). The red arrow indicates the location of the target bands

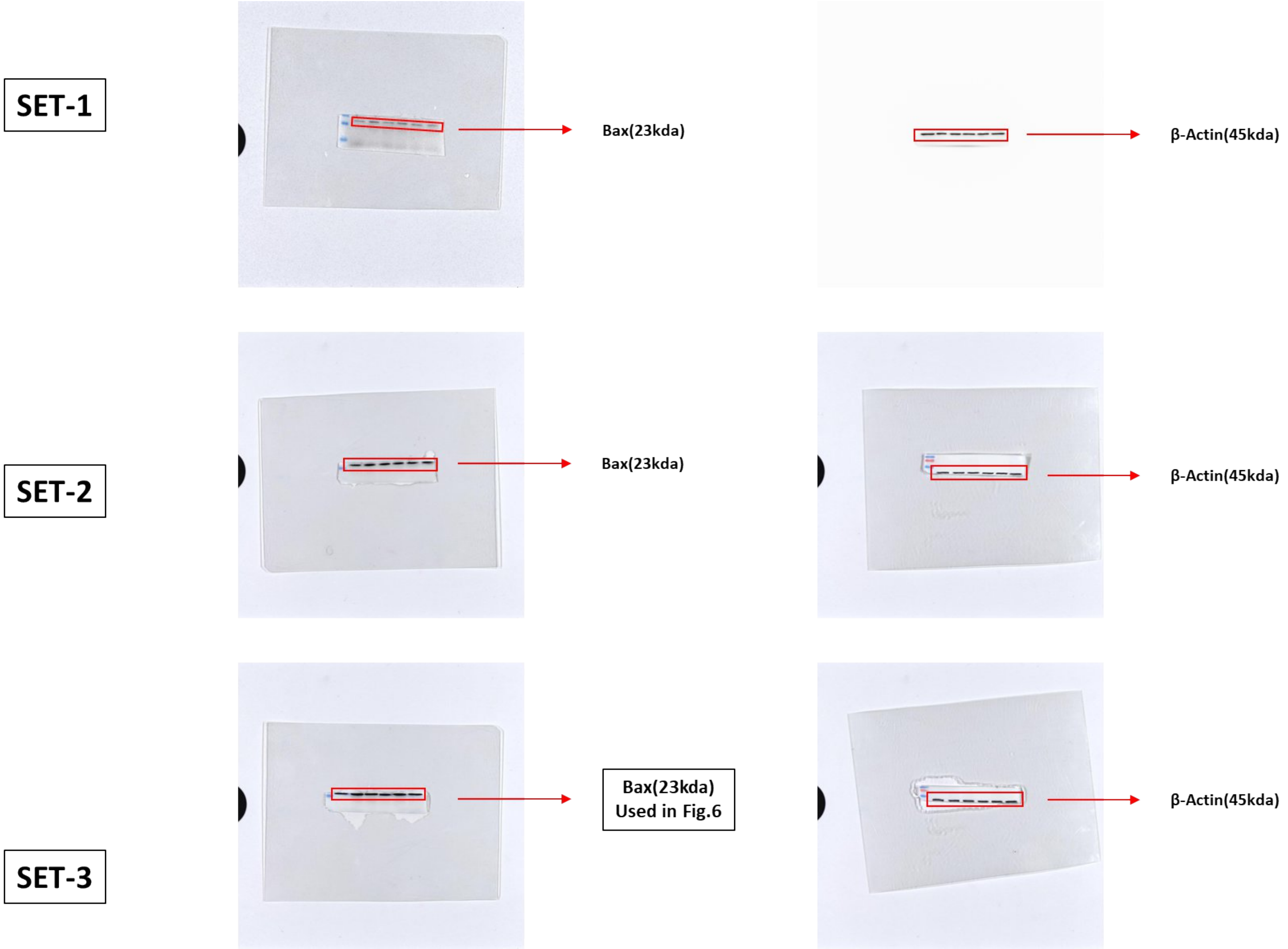

Supplementary Figure 15: The Uncropped immune blot data of Bcl-2 in I/R-induced kidney (Figure 6). The red arrow indicates the location of the target bands

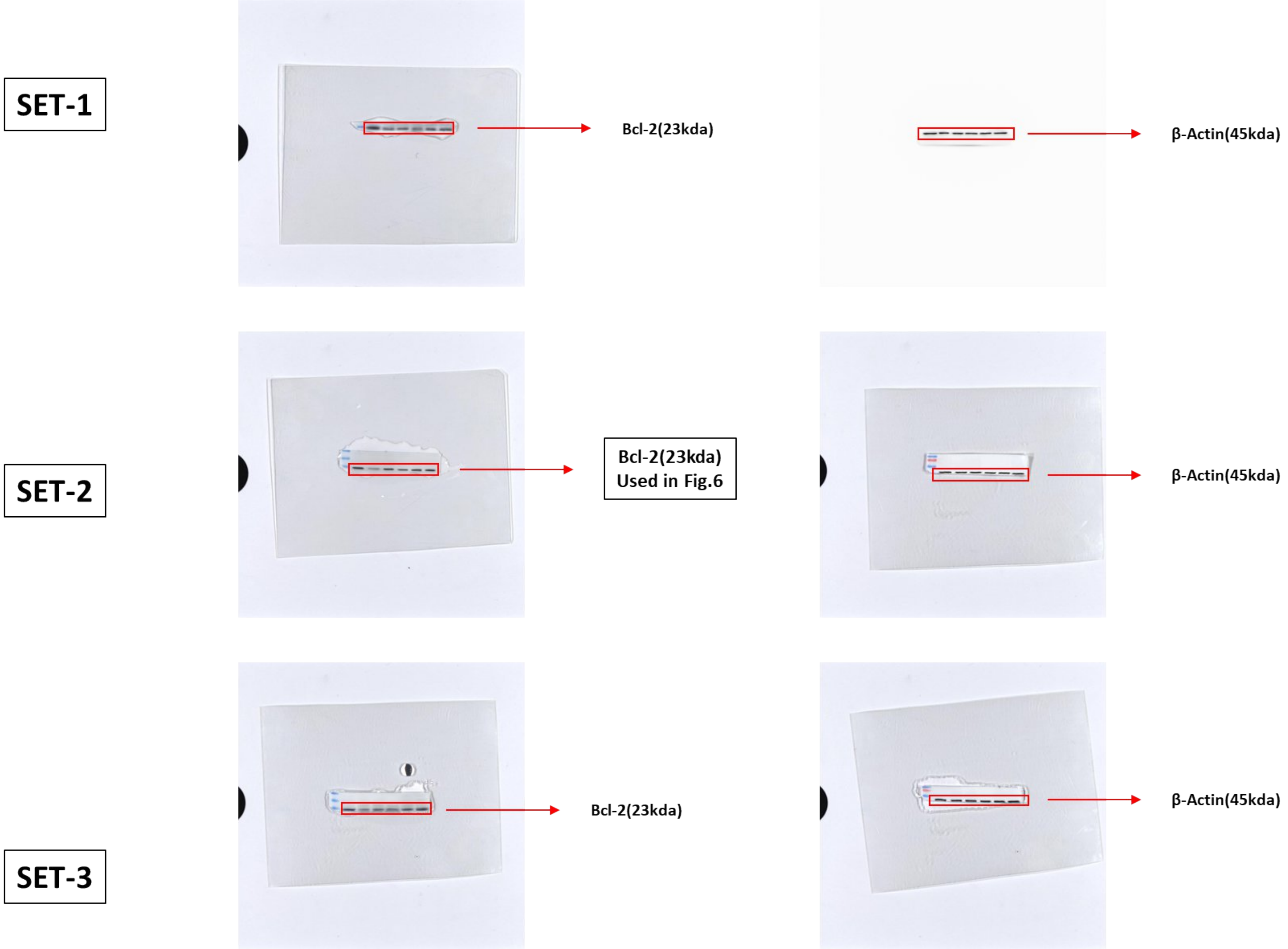

Supplementary Figure 16: The Uncropped immune blot data of C-Cas-3 in I/R-induced kidney (Figure 6). The red arrow indicates the location of the target bands

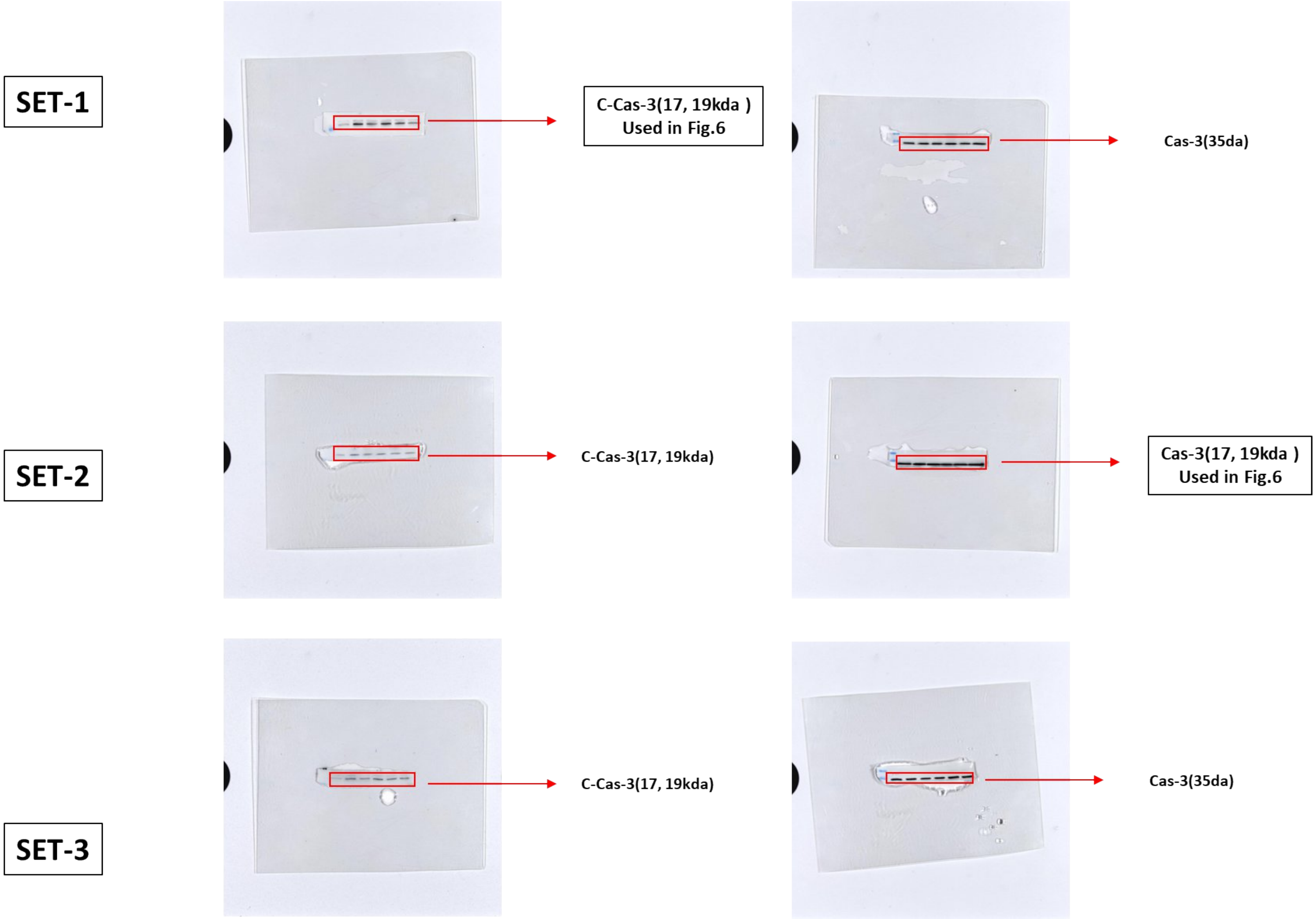

Supplementary Figure 17: The Uncropped immune blot data of TNF-α in I/R-induced kidney (Figure 5). The red arrow indicates the location of the target bands

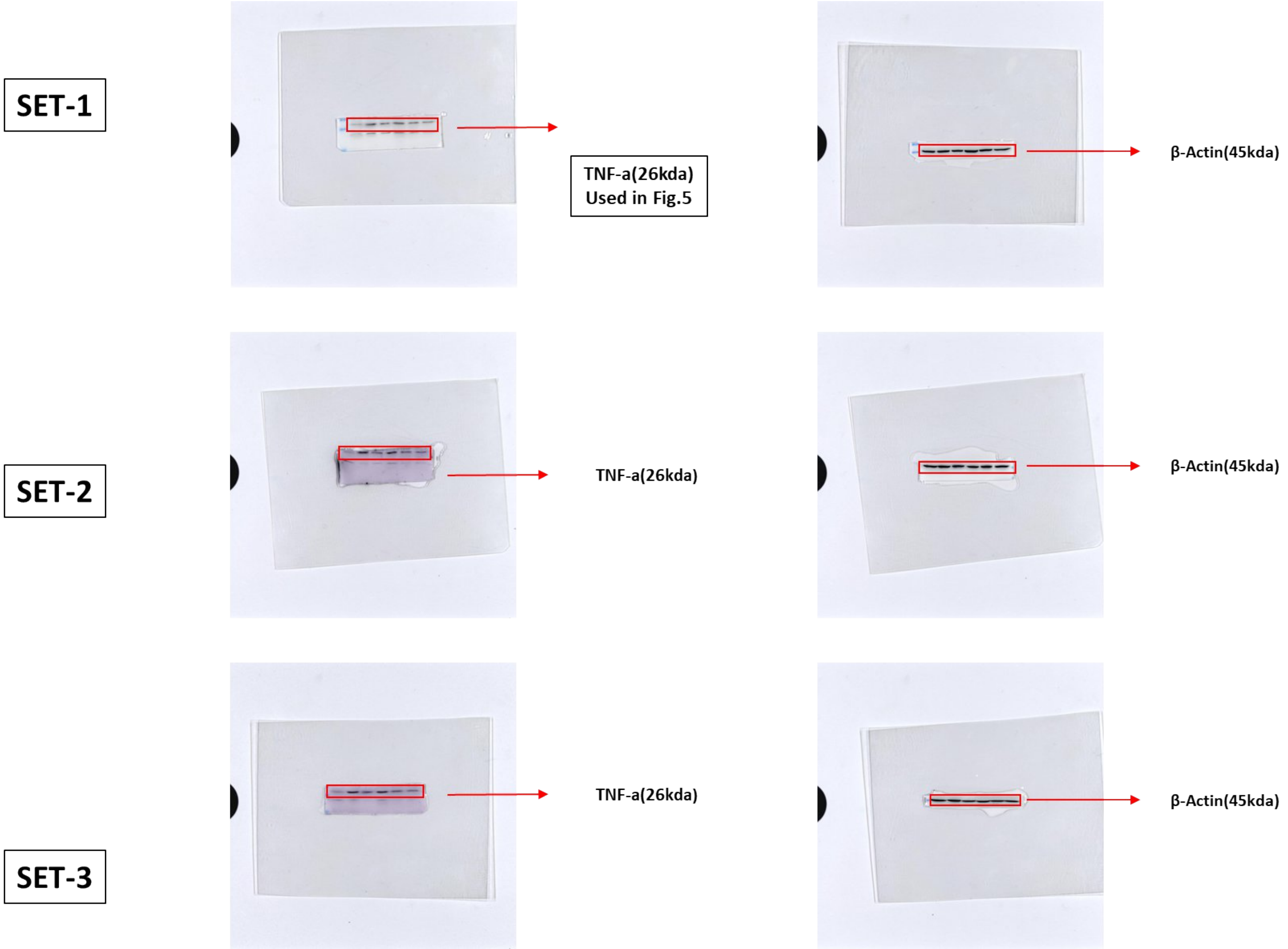

Supplementary Figure 18: The Uncropped immune blot data of IL-1β in I/R-induced kidney (Figure 5). The red arrow indicates the location of the target bands

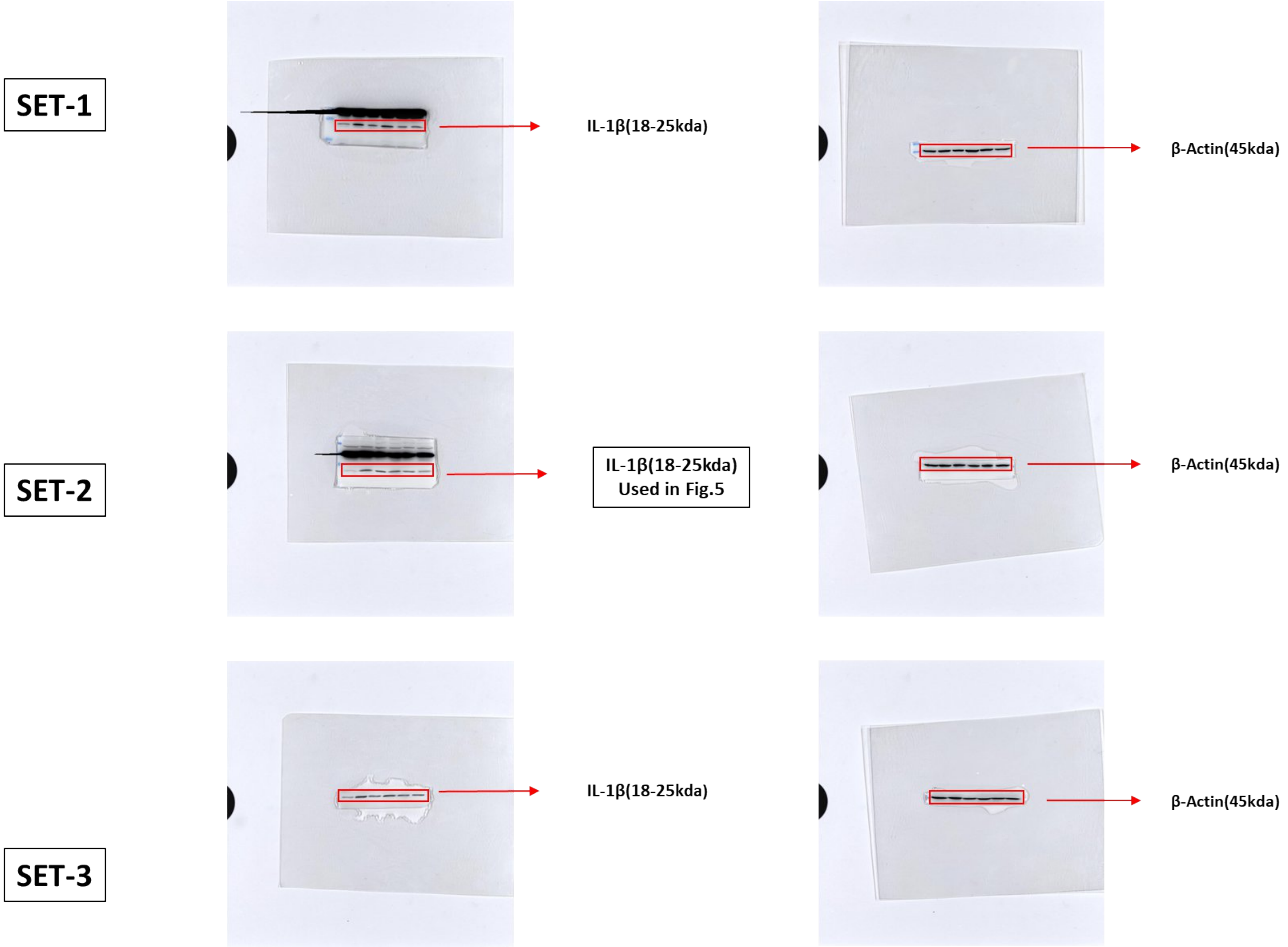

Supplementary Figure 19: The Uncropped immune blot data of p-NF-κB and NF-κB in I/R-induced kidney (Figure 5). The red arrow indicates the location of the target bands

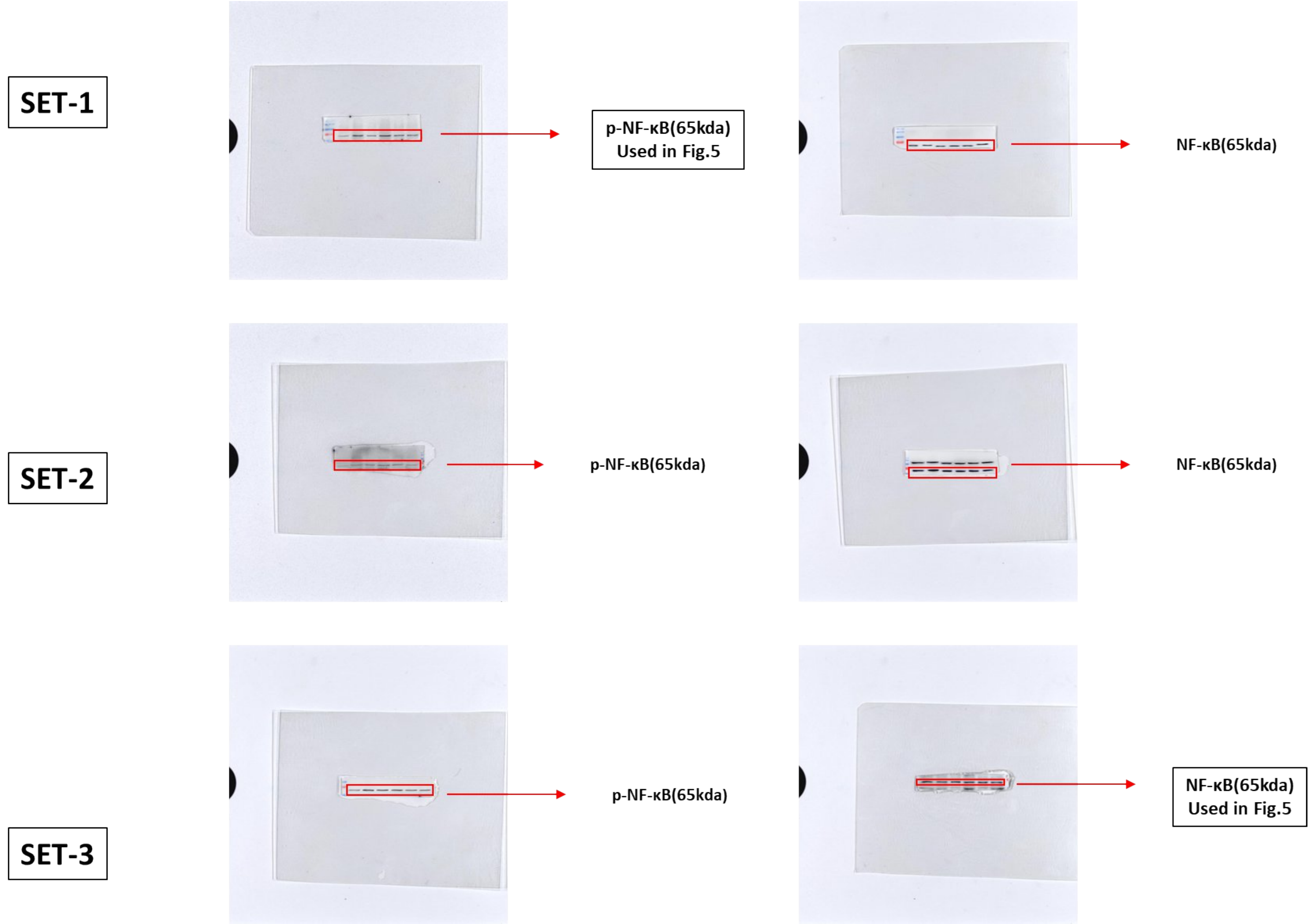

Supplement: Multimedia component 2 [file mmc2.pdf]
